# The anti-apoptotic function of HSV-1 LAT in neuronal cell cultures but not its function during reactivation correlates with expression of two small non-coding RNAs, sncRNA1&2

**DOI:** 10.1371/journal.ppat.1012307

**Published:** 2024-06-10

**Authors:** Jay J. Oh, Ujjaldeep Jaggi, Kati Tormanen, Shaohui Wang, Satoshi Hirose, Homayon Ghiasi

**Affiliations:** Center for Neurobiology and Vaccine Development, Ophthalmology Research, Department of Surgery, Cedars-Sinai Burns & Allen Research Institute, CSMC–SSB3, Los Angeles, California, United States of America; Oklahoma State University College of Veterinary Medicine, UNITED STATES

## Abstract

Multiple functions are associated with HSV-1 latency associated transcript (LAT), including establishment of latency, virus reactivation, and antiapoptotic activity. LAT encodes two sncRNAs that are not miRNAs and previously it was shown that they have antiapoptotic activity *in vitro*. To determine if we can separate the antiapoptotic function of LAT from its latency-reactivation function, we deleted sncRNA1 and sncRNA2 sequences in HSV-1 strain McKrae, creating ΔsncRNA1&2 recombinant virus. Deletion of the sncRNA1&2 in ΔsncRNA1&2 virus was confirmed by complete sequencing of ΔsncRNA1&2 virus and its parental virus. Replication of ΔsncRNA1&2 virus in tissue culture or in the eyes of WT infected mice was similar to that of HSV-1 strain McKrae (LAT-plus) and dLAT2903 (LAT-minus) viruses. The levels of gB DNA in trigeminal ganglia (TG) of mice latently infected with ΔsncRNA1&2 virus was intermediate to that of dLAT2903 and McKrae infected mice, while levels of LAT in TG of latently infected ΔsncRNA1&2 mice was significantly higher than in McKrae infected mice. Similarly, the levels of LAT expression in Neuro-2A cells infected with ΔsncRNA1&2 virus was significantly higher than in McKrae infected cells. Reactivation in TG of ΔsncRNA1&2 infected mice was similar to that of McKrae and time of reactivation in both groups were significantly faster than dLAT2903 infected mice. However, levels of apoptosis in Neuro-2A cells infected with ΔsncRNA1&2 virus was similar to that of dLAT2903 and significantly higher than that of McKrae infected cells. Our results suggest that the antiapoptotic function of LAT resides within the two sncRNAs, which works independently of its latency-reactivation function and it has suppressive effect on LAT expression *in vivo* and *in vitro*.

## Author summary

The LAT region encodes two small non-coding RNAs (LAT sncRNA1 and sncRNA2) and they are expressed in TG of latently infected mice. The sncRNA1 and sncRNA2 are not miRNAs and they can inhibit apoptosis and promote cell survival *in vitro*. sncRNA1 and sncRNA2 are expressed from the first 1.5-kb of LAT coding sequences and the reactivation and antiapoptotic activity of LAT is within the 1.5 kb of LAT. To determine the role of sncRNA1&2 on latency-reactivation and apoptosis, we constructed a recombinant virus lacking both sncRNA1&2 (i.e., ΔsncRNA1&2). This recombinant virus grow in tissue culture and in the eyes of infected mice similar to that of control viruses. Here we demonstrated a novel function for sncRNA1&2 in reducing antiapoptotic function of LAT and down-regulation of LAT expression *in vitro* and *in vivo*. We have also shown that the two sncRNAs encoded within the 1.5 kb of LAT do not play a role in virus reactivation.

## Introduction

The herpes simplex virus type 1 (HSV-1) is one of the most well-adapted human pathogens [1,2]. The infection of HSV-1 takes place at epithelial and mucosal surfaces, where the virus actively replicates and form lesions that involves the combined interaction of innate and adaptive immunity [3,4]. Following primary infection, the virus travels sensory nerve endings and enters in axons of the peripheral nervous system (PNS) and establishes a lifelong latent infection in sensory neurons located in dorsal root ganglia or trigeminal ganglia (TG) [5–7]. Previously we and others have shown presence of LAT expression in the neurons of infected host (i.e., mice, rabbits, and humans) [5,6,8,9]. The latency-related RNA (LR-RNA) was originally reported by Dan Rock [10] and later on renamed as the latency-associated transcript (LAT) [11]. LAT is the only gene product consistently detected in abundance during latency in infected host [5,6,8,9]. The LAT product is present in two copies per genome and the primary LAT product is 8.3 kb and is unstable, while a very stable 2 kb LAT is derived from the primary transcript by splicing [6,9,12–14]. In addition to the 2 kb LAT, a second LAT RNA of 1.3–1.5-kb is also abundant during latency and is derived from the 2 kb LAT by splicing [10,15].

LAT does not express any protein during primary infection or latency and is anti-sense to ICP0 [10]. It is not necessary for initial infection or virulence [16–18], however as an RNA molecule, LAT plays a significant function during the lifetime of the infected host such as: 1) higher detection of viral DNA and LAT during latency in the presence of LAT [16,19,20]; 2) higher rate of *in vivo* spontaneous and induced reactivation from latency in the presence of LAT [8,16]; 3) apoptosis is reduced in the presence of LAT both *in vivo* and *in vitro* [21–26]; 4) LAT contributes to CD8^+^ T cell exhaustion [19,20]; 5) LAT downregulates components of the Type I Interferon (IFN) pathway [27]; 6) LAT also interferes with expressions of ICP0 and ICP4 in infected cells [28, 29]; and 7) LAT activates HVEM to enhance latency [30].

The LAT region encodes micro-RNAs (miRNA) [31–33]. In addition, two small non-coding RNAs (sncRNAs), 62 nt and 36 nt long, are expressed from the first 1.5-kb of LAT coding sequences (LAT sncRNA1 and sncRNA2) and are expressed in TG of latently infected mice [34]. Expression of the first 1.5-kb of LAT coding sequence is crucial for reactivation of the HSV-1 in rabbit models [23,25]. The antiapoptotic activity of LAT is within the 1.5 kb LAT that has been spliced from the 2 kb stable LAT [6,9,21]. The LAT sncRNA1 and sncRNA2 are not miRNAs because the mature miRNA band that migrates between 21 and 23 nucleotides is not detected, they lack certain structural features of miRNAs, and both sncRNAs have the potential to form complex secondary structures. These two sncRNAs can inhibit apoptosis and promote cell survival, reduce the efficiency of productive infection, interact with retinoic acid inducible gene I (RIG-I), enhance IFNβ promoter activity, and increase HVEM activity [30,35]. On transient transfection, LAT sncRNAs inhibits cold shock induced apoptosis in mouse neuroblastoma cells and productive infection [36]. Expressions of sncRNA1 and 2 inhibits apoptosis and productive infection *in vitro* [36] and they activate HVEM promoter activity *in vitro* [37].

Recently we reported construction of a recombinant virus lacking the 62 bp sncRNA1 sequence in HSV-1 strain McKrae and creating recombinant virus ΔsncRNA1 [38]. Replication of ΔsncRNA1 virus in tissue culture or in the eyes of infected mice was similar to that of HSV-1 strain McKrae and dLAT2903 viruses. However, the absence of sncRNA1 significantly reduced expression levels of ICP0, ICP4, and gB, but not LAT transcript in infected rabbit skin cells *in vitro*. By contrast, the absence of sncRNA1 reduced LAT expression in trigeminal ganglia (TG), but not in corneas, by day 5 post infection (PI) in infected mice. Levels of eye disease in mice infected with ΔsncRNA1 and McKrae viruses were similar, and despite reduced LAT levels in TG during acute ΔsncRNA1 infection, McKrae and ΔsncRNA1 viruses did not affect latency or reactivation on day 28 PI.

In this study, we sought to determine what role if any the absence of both sncRNA1&2 may have on HSV-1 infectivity *in vitro* and *in vivo* as well as contribution of these two sncRNAs may have on HSV-1 latency-reactivation and apoptosis. Thus, we constructed a recombinant virus lacking both sncRNA1&2 (i.e., ΔsncRNA1&2) and confirmed the absence of both sncRNA1&2 by complete sequencing of ΔsncRNA1&2 virus and compared this recombinant virus with its parental dLAT2903 (LAT-minus) and WT parental McKrae (LAT-plus) viruses *in vitro* and *in vivo*. Our results suggest that: 1) The kinetics of ΔsncRNA1&2 virus replication *in vitro* and in the eyes of infected mice were similar to that of McKrae and dLAT2903 viruses; 2) Survival, corneal scarring and angiogenesis of ΔsncRNA1&2 virus were similar to control viruses; 3) Levels of gB DNA in TG of latently infected ΔsncRNA1&2 was intermediate to that of McKrae (LAT-plus) and dLAT2903 (LAT-minus), while LAT expression levels were higher in TG of ΔsncRNA1&2 mice compared with McKrae infected mice and similar results were obtained in Neuro-2A-infected cells; 4) Reactivation was the same in TG of latently infected ΔsncRNA1&2 mice with that of WT McKrae and both were faster than in dLAT2903 infected TG; and 5) Levels of apoptosis in Neuro-2A cells infected with ΔsncRNA1&2 virus was similar to that of dLAT2903 and both were significantly higher than McKrae infected cells. Overall, in this study we have shown that similar to the LAT-minus virus, the antiapoptotic function of LAT is located within the two sncRNA1&2 regions of LAT and in contrast to LAT-minus virus they are not playing any role in virus reactivation.

## Results

### Structure of ΔsncRNA1&2

**Fig 1:**
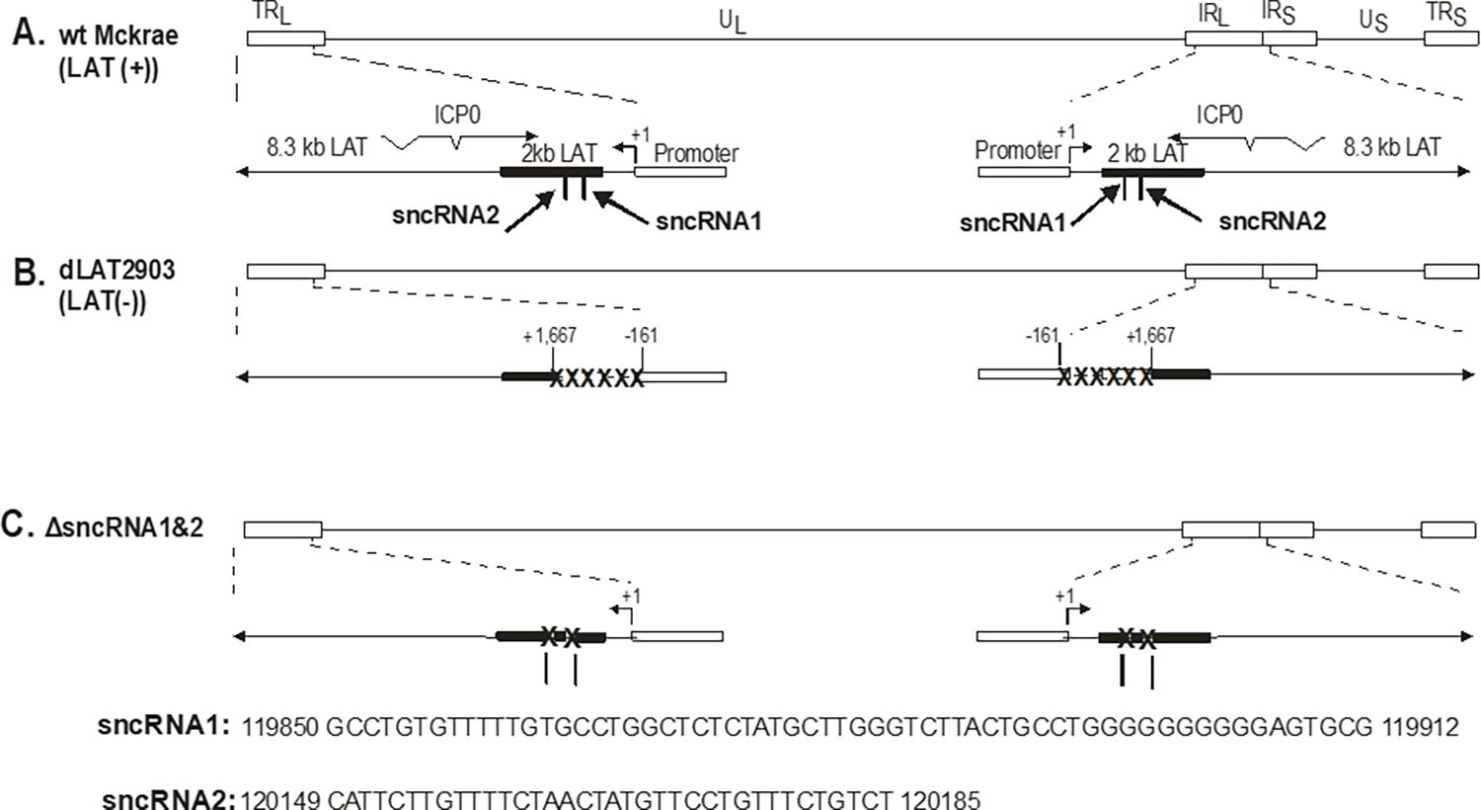
Construction and structure of the ΔsncRNA1&2 recombinant virus. **(A)** Diagram of WT HSV-1 genome showing locations of ICP0, 8.3kb primary LAT, 2kb stable LAT loci in the long terminal repeat and internal long repeat, and location of sncRNA1 and sncRNA2 in the long terminal repeat and internal long repeat; **(B)** Diagram of dLAT2903 depicting the removed LAT regions including portion of the LAT promoter and downstream sequences of the stable 2kb LAT; and **(C)** Diagram of ΔsncRNA1&2 showing restoration of all but the sncRNA1 and sncRNA2 sequences in the stable 2kb LAT and its promoter sequences. The missing 62 bp and 36 bp sncRNA1 and sncRNA2 sequences, respectively are shown. TR_L_: Terminal repeat long; U_L_: Unique long; IR_L_: Internal repeat long; IR_S_: Internal repeat short; U_S_: Unique short; and TR_S_: Terminal repeat short.

HSV-1 strain McKrae was used as the original parental virus and its genomic structure is shown schematically in Fig 1A. The HSV-1 genome contains a unique long region (U_L_) and a unique short region (U_S_) (solid lines), each flanked by inverted repeats (open rectangles); terminal and internal repeats long (TR_L_ and IR_L_) and terminal and internal repeats short (TR_S_ and IR_S_). The location of the LAT promoter TATA box is indicated as TATA. The LAT transcription start site is 28 nt downstream of the TATA box [39]. The LAT-null mutant dLAT2903, containing a 1.8-kb deletion in both copies of the LAT gene (one in each long repeat) [16] is shown schematically in Fig 1B. This deletion (indicated by XXXX) consists of 0.2 kb of the LAT promoter and a portion of the LAT gene encoding the first 1.6-kb of the 8.3-kb primary LAT transcript. ΔsncRNA1&2 virus was derived from dLAT2903 by inserting the 1.8-kb LAT lacking sncRNA1 and sncRNA2 sequences (Fig 1C) as described in Materials and Methods. As confirmed by restriction enzyme analysis and whole genome sequencing (gene bank, accession id OR723971), the ΔsncRNA1&2 virus contains the complete region of LAT similar to WT HSV-1 strain McKrae but without the 62 bp and the 36 bp regions of sncRNA1 and sncRNA2, respectively (Fig 1C). The whole genome sequencing of ΔsncRNA1&2 virus confirmed absence of sncRNA1 sequence (5’-GCCTGTGTTTTTGTGCCTGGC TCTCTATGCTTGGGTCTTACTGCCTGGGGGGGGGGAGTGCG-3’) at position 119,887–199,948 on positive strand (S1A Fig) and 6,476–6,537 on negative strand (S1B Fig), and absence of sncRNA2 sequence (5’-CATTCTTGTTTTCTAACTATGTTCCTGTTTCTGTCT-3) at position 120,280–120,315 on positive strand (S1C Fig) and 6,109–6,144 on negative strand (S1D Fig). We did not detect any differences between ΔsncRNA1&2 virus with WT HSV-1 strain McKrae (gene bank, accession id OR723971).

### Absence of sncRNA1 and 2 sequences in ΔsncRNA1&2 virus does not alter virus replication *in vitro* compared with control viruses

**Fig 2:**
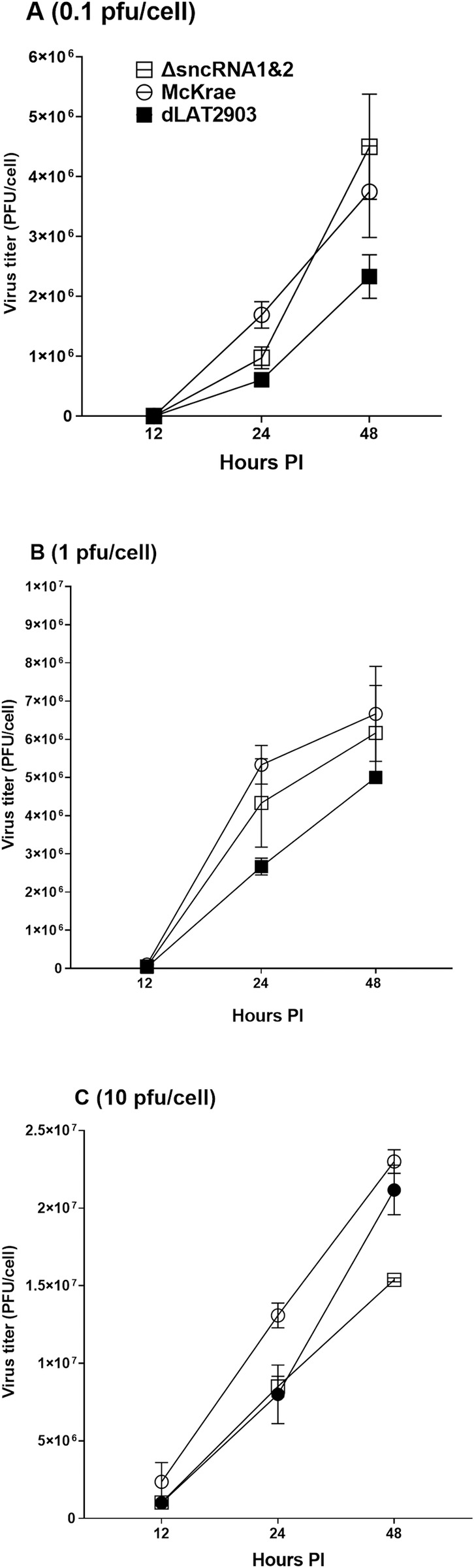
Loss of sncRNA1&2 sequences does not affect viral replication *in vitro*. RS cells were infected with ΔsncRNA1&2, WT McKrae, or dLAT2903 virus at **(A)** 0.1 pfu/cell, **(B)** 1 pfu/cell, or **(C)** 10 pfu/cell for 12, 24, or 48 hr. Titers of each virus at each time point were determined using standard plaque assay. No significant differences in titer between the three viruses were seen at any infectious dose or timepoint. Results are shown as mean ± SEM from two separate experiments (n = 6).

To determine if the absence of sncRNA1 and 2 sequences in ΔsncRNA1&2 virus, affects virus replication *in vitro*, RS cells were infected with 0.1, 1, or 10 pfu/cell of WT McKrae, dLAT2903, or ΔsncRNA1&2 virus for 12, 24, or 48 hr. Virus titers in infected cells were determined by standard plaque assay. There were no statistically significant differences amongst the three viruses at any pfu or time point (Fig 2A-2C, p>0.05). These results demonstrate that deletion of the sncRNA1 and sncRNA2 sequences does not affect virus replication *in vitro*.

### Virus replication in the eyes of ΔsncRNA1&2 virus infected mice is not affected by the absence of sncRNA1&2 sequences

**Fig 3:**
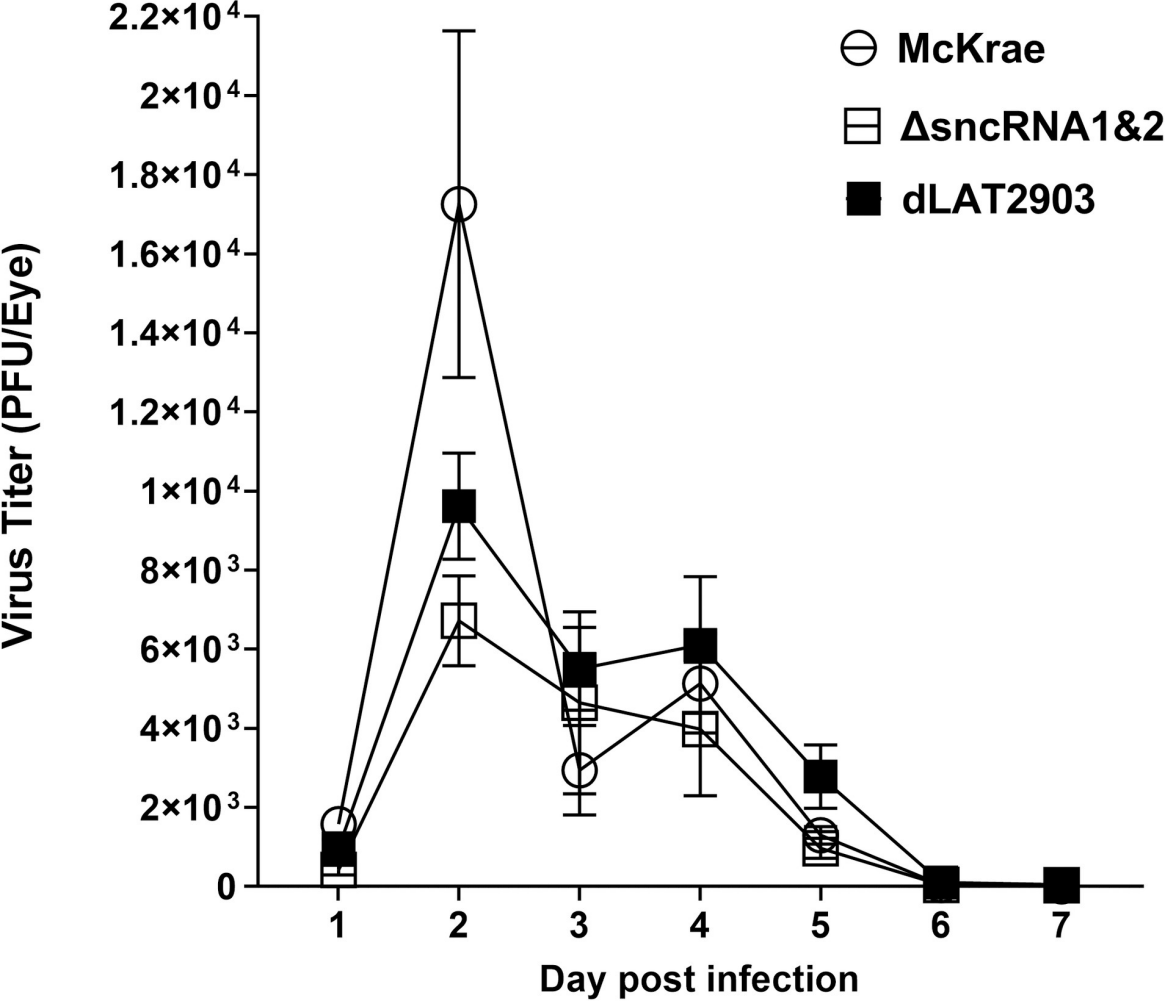
Loss of sncRNA1&2 sequences does not affect viral replication in the eye of infected mice. Mice were infected ocularly with 2 X 10^5^ pfu/eye of WT McKrae, dLAT2903, or ΔsncRNA1&2 virus without corneal scarification. Tear films were collected on days 1–7 PI and virus titers determined by standard plaque assay. The experiment was repeated twice, and each point represents the mean ± SEM (Fisher’s exact test) from 20 eyes per group.

To determine if sncRNA1&2 affects HSV-1 replication during primary ocular infection, WT mice were ocularly infected with 2 X 10^5^ pfu/eye of HSV-1 McKrae, dLAT2903, or ΔsncRNA1&2 virus as described in Materials and Methods. Tears were collected from 40 eyes/group from two separate experiments on days 1 to 7 post-infection (PI). The amount of virus in each eye was determined using a standard plaque assay. Virus titers were similar in all three groups of infected mice on all days, suggesting that the sncRNA1&2 sequences of LAT are not necessary for virus replication *in vivo* (Fig 3, p>0.05). This is similar to previous studies showing the absence of the entire 2kB LAT does not affect virus replication in the eyes of infected mice and rabbits [16,19,20,40].

### The absence of sncRNA1&2 does not affect survival and eye disease in ΔsncRNA1&2 infected mice compared with control viruses

**Fig 4:**
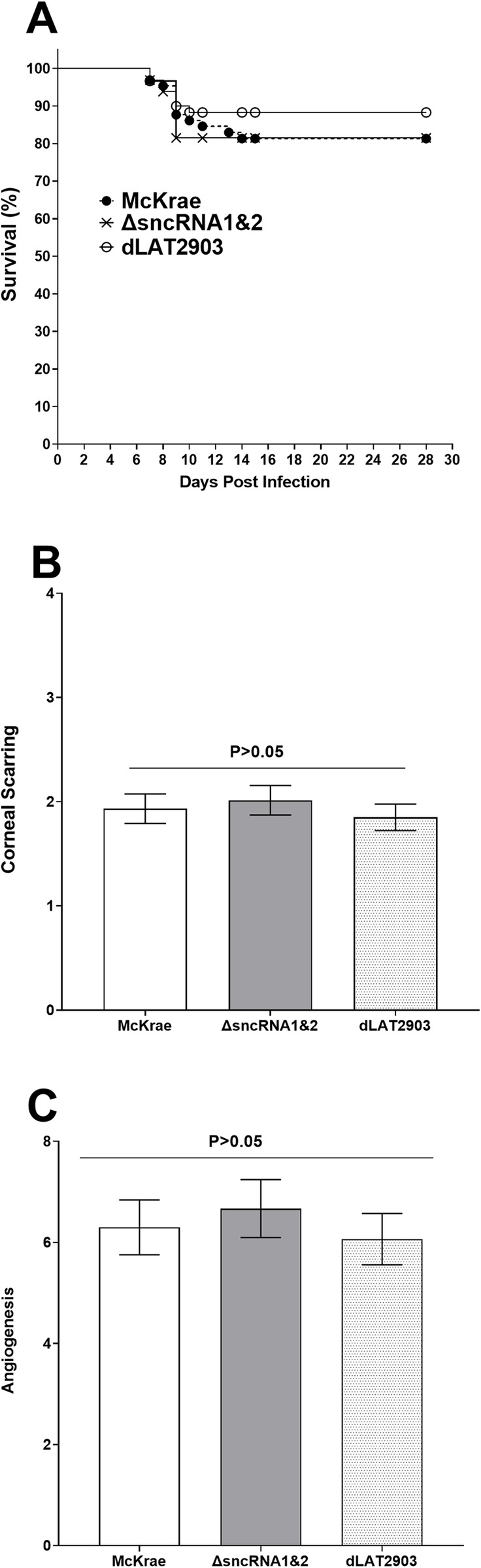
Loss of sncRNA1&2 sequences does not affect mouse survival, corneal scarring (CS) or angiogenesis in ocularly infected mice. (A) Survival of ocularly infected mice. Mice were ocularly infected with 2 X 10^5^ pfu/eye of ΔsncRNA1&2, WT McKrae or dLAT2903. Survival of infected mice was monitored for 28 days PI. Graph represents the average of six independent experiments with 60 mice per virus strain (Chi squared test); and (B-C) CS and angiogenesis in surviving mice. CS and angiogenesis in surviving mice described above were examined on day 28 PI as described in Materials and Methods. The CS and angiogenesis scores represent the average ± SEM from 96 eyes for ΔsncRNA1&2 and WT McKrae viruses and 106 eyes for dLAT2903 virus.

Survival of mice infected with McKrae, dLAT2903 and ΔsncRNA1&2 viruses from six independent experiments and sixty mice per virus strain was monitored for 28 days and survival data from three separate experiments were combined. Forty-eight of 60 (80% survival) mice infected with ΔsncRNA1&2 virus survived ocular infection, similar number of mice (48/60; 80% survival) infected with McKrae survived ocular infection, while fifty-three of sixty (88% survival) mice infected with dLAT2903 survived ocular infection (Fig 4A). These differences amongst three groups of infected mice were not statistically significant (Fig 4A, p>0.05; Fisher exact test). These results suggest that the absence of sncRNA1&2 did not affect neurovirulence in infected mice. Corneal scarring and angiogenesis in surviving mice described above (Fig 4A) was scored in a blinded fashion on day 28 PI as described in Materials and Methods. There were no differences in either CS (Fig 4B; p>0.05) or angiogenesis (Fig 4C; p>0.05) amongst mice infected with WT McKrae, ΔsncRNA1&2, and dLAT2903 viruses, suggesting that the sncRNA1&2 sequences does not alter eye disease in ocularly infected WT mice. This is similar to previous studies showing the absence of the entire 2kB LAT does not affect eye disease compared with WT HSV-1 strain McKrae virus [19,41,42].

### Absence of sncRNA1&2 does not alter reactivation compared with WT virus

**Fig 5:**
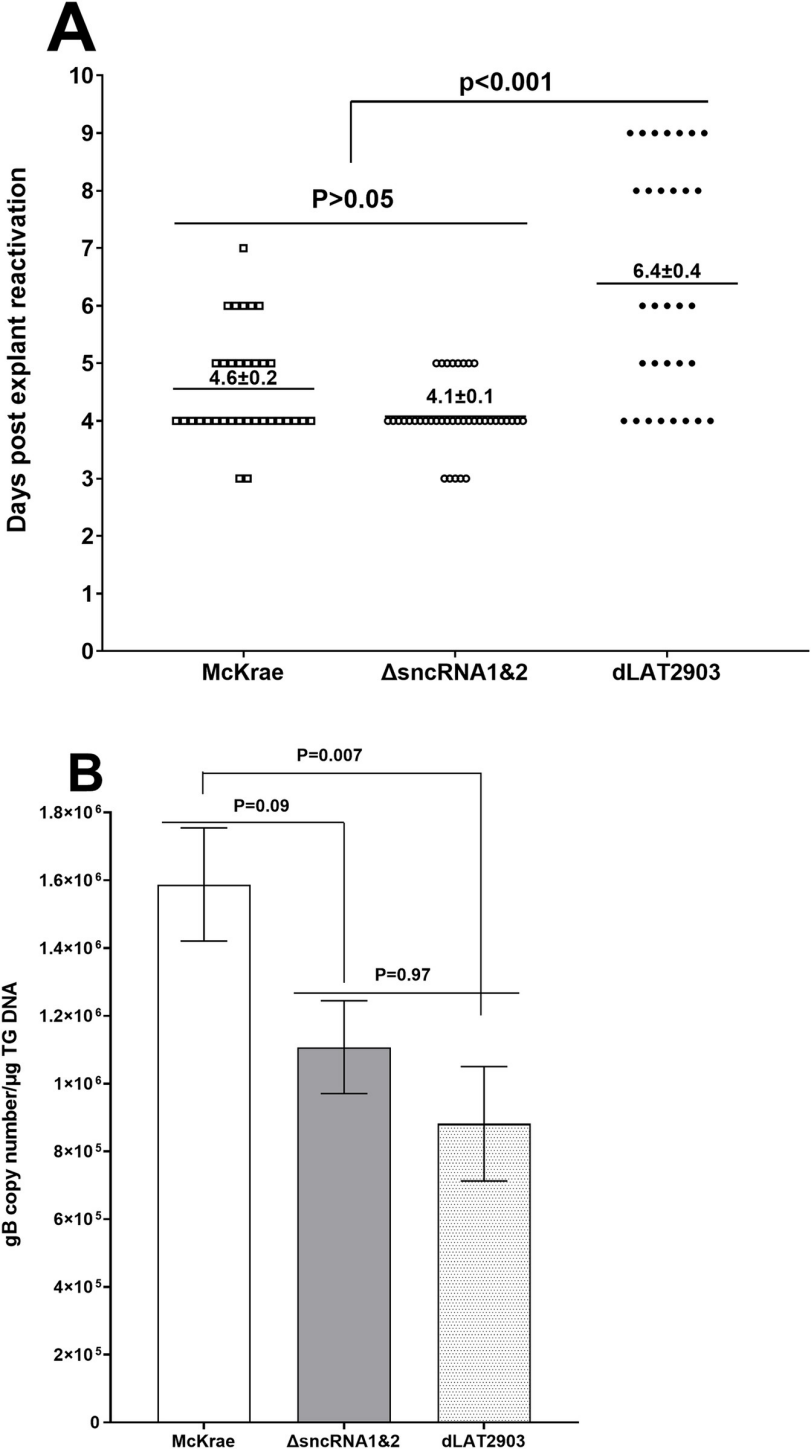
Effect of absence of sncRNA1&2 on reactivation and gB DNA. Mice were infected ocularly as above with ΔsncRNA1&2, McKrae or dLAT2903 virus. TG were harvested on day 28 PI. (A) Explant reactivation in TG of latently infected mice. TG from mice described above were harvested and time to reactivation was determined by explant reactivation assay. Points represent day at which CPE was first observed. Horizontal line is mean time to reactivation ± SEM from 42 TG for ΔsncRNA1&2, 38 TG for McKrae and 36 TG for dLAT2903. Experiment was repeated twice; and (B) gB copy number in TG of latently infected mice. DNA isolated from individual TG described above was used to determine relative gB copy number by qPCR as described in Materials and Methods. Bars represent mean gB copy number ± SEM from 30 TG from ΔsncRNA1&2 infected mice; 32 TG for McKrae infected mice, and 28 TG from dLAT2903 infected mice. Experiment was repeated twice.

Previously we reported that reactivation in dLAT2903 infected mice was significantly slower than McKrae infected animals [16,19,20,40]. To determine if the absence of sncRNA1&2 affects time to reactivation in ocularly infected mice, a group of 25 mice from three separate experiments were ocularly infected with WT McKrae, ΔsncRNA1&2, and dLAT2903 viruses as described above. Virus reactivation was analyzed by explanting individual TG from infected mice on day 28 PI as described in Materials and Methods and time to reactivation in explant TG was monitored for 10 days post TG culture. In general, time to reactivation in ΔsncRNA1&2 infected mice was faster than McKrae infected mice but the average time to reactivation between McKrae and ΔsncRNA1&2 groups was not statistically significant (4.6±0.2 days for McKrae and 4.1±0.1 days for ΔsncRNA1&2 infected mice) (Fig 5A, p>0.05; Fisher exact test). However, despite the absence of significant differences in average time to reactivation between McKrae and ΔsncRNA1&2 groups, in ΔsncRNA1&2 group, time of reactivation was faster (i.e., days 3–5) compared with time of reactivation in TG from McKrae group (i.e., days 3–7). Consistent with previous studies [16,19,20,40], time to reactivation in dLAT2903 infected mice was significantly delayed compared to McKrae and ΔsncRNA1&2 viruses (Fig 5A; dLAT2903; 6.4 ± 0.4 days vs McKrae and ΔsncRNA1&2; *p*<0.001). Overall, by day 10 post explant reactivation monitoring, in ΔsncRNA1&2 infected TG, 39 of 42 TG reactivated, in McKrae infected TG 34 of 38 reactivated, while in dLAT2903 infected TG 31 of 36 TG reactivated. Thus, in contrast to the absence of LAT in dLAT2903 virus that reduced time of explant reactivation, the absence of sncRNA1&2 did not alter to explant reactivation in ΔsncRNA1&2 infected mice. These results suggest that LAT sequences outside of the sncRNA1&2 are sufficient for WT reactivation phenotype.

### Effect of sncRNA1&2 absence on gB expression during latency

Reactivation results described above (Fig 5A), suggested that despite non-significant but faster reactivation time in ΔsncRNA1&2 infected TG were similar to McKrae-infected TG. Thus, to determine if the absence of sncRNA1&2 affects levels of latency, in two separate experiments, mice were ocularly infected with WT McKrae, ΔsncRNA1&2, and dLAT2903 viruses as above. Since dLAT2903 lacking 2 kb LAT, levels of gB DNA in latently infected TG was determined by qPCR using 32 TG for McKrae, 30 TG for ΔsncRNA1&2 virus and 28 TG for dLAT2903 virus on day 28 PI. Consistent with our previous reports [20,43], there was significantly more HSV-1 DNA in TG from mice latently infected with McKrae compared to dLAT2903 virus (Fig 5B; McKrae vs dLAT2903; *p* = 0.007) characteristics of more latency with McKrae compared to dLAT2903. However, levels of gB DNA in TG of ΔsncRNA1&2 infected mice was intermediate to that of McKrae and dLAT2903 groups (Fig 5B). Levels of gB DNA in TG of ΔsncRNA1&2 mice was similar to that of dLAT2903 (Fig 5B; ΔsncRNA1&2 vs dLAT2903; p = 0.97), while levels of gB DNA in ΔsncRNA1&2 infected TG was approximately 30% lower than McKrae infected TG but these differences were not statistically significant using ANOVA (Fig 5B, p = 0.09). Thus, as expected dLAT2903 infected mice had significantly fewer latent viral genomes than did McKrae infected mice, while the absence of sncRNA1&2 reduced levels of latency similar to that of dLAT2903 but lower than McKrae infected TG.

### LAT expression during latency is upregulated in the absence sncRNA1&2

**Fig 6:**
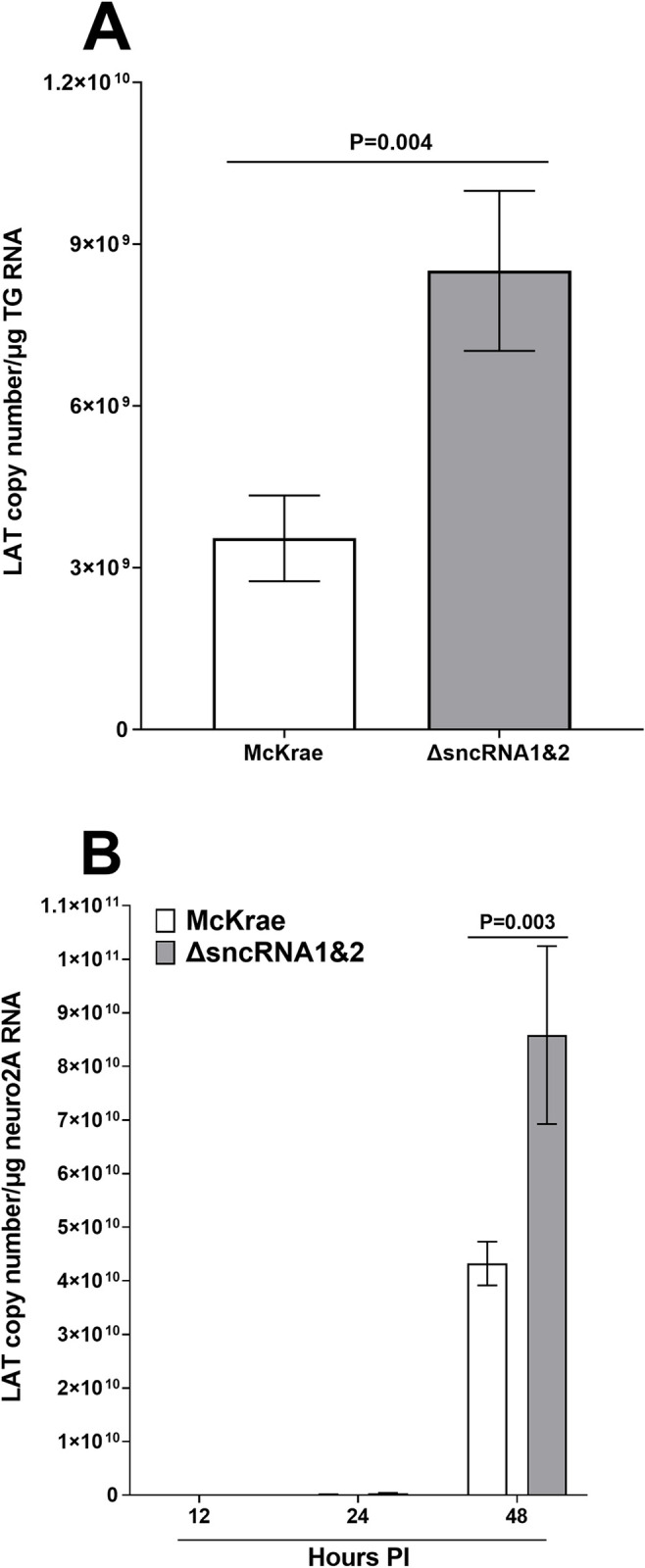
Effect of absence of sncRNA1&2 on LAT expression *in vivo* and *in vitro*. (A) LAT expression in TG of latently infected mice. Mice were ocularly infected with 2 X 10^5^ pfu/eye of WT McKrae or ΔsncRNA1 virus as above. Ocularly infected mice were euthanized on day 28 PI and TG were collected. Total RNA was extracted from collected tissues and relative LAT RNA copy number was quantified as described in Materials and Methods. Bars represent mean LAT copy number ± SEM from 26 TG for ΔsncRNA1&2 infected mice and 30 TG for McKrae infected mice. Experiment was repeated twice; and (B) LAT expression in Neuro-2A infected cells. Neuro-2A cells were grown to confluency and infected with 0.1 pfu/cell of McKrae or ΔsncRNA1&2 virus for 12, 24 and 48 hr. At 12, 24, and 48 hr PI, cells were harvested, washed, total RNA was isolated, and RT-PCR on isolated RNA was performed as above. Bars represent mean LAT copy number ± SEM (N = 3).

Levels of gB DNA in TG of ΔsncRNA1&2 infected mice described above was lower but not significant compared with TG of McKrae infected mice (Fig 5B). Thus, we next determined if absence of sncRNA1&2 affects levels of LAT expression in TG of latently infected mice. Mice were ocularly infected with WT McKrae or ΔsncRNA1&2 virus as above and experiments were repeated three times. Total RNA from 50 TG of WT McKrae and 49 TG of ΔsncRNA1&2 virus were extracted on day 28 PI and LAT copy numbers were measured by qRT-PCR. In contrast to gB DNA described above (Fig 6A), mice infected with ΔsncRNA1&2 virus had significantly higher LAT expression compared with WT McKrae (Fig 6A). These differences were highly significant (Fig 6A; P = 0.004; Fisher exact test). The RT-PCR results for LAT suggest that the absence of sncRNA1&2 upregulated expressions of LAT RNA but not gB DNA in latently infected TG. Thus, presence of sncRNA1&2 within the LAT has an inhibitory effect on LAT expression *in vivo*. Although, since levels of LAT correlate with faster reactivation and more pathology, virus may use these sncRNAs as a mechanism of self-protection.

The above results discussed in Fig 6A suggested that the two sncRNAs have an inhibitory effect on LAT expression *in vivo*. To confirm if LAT upregulation by ΔsncRNA1&2 virus can occur *in vitro*, Neuro-2A cells were infected with 0.1 pfu/cell of ΔsncRNA1&2 and McKrae viruses for 12, 24 and 48 hr as described in Materials and methods. Total RNA from infected cells were isolated and qRT-PCR was performed. No detectable levels of LAT were detected for both viruses at 12 and 24 hr PI, while levels of LAT expression at 48 hr PI increased for both groups and was significantly higher in ΔsncRNA1&2 infected group than in McKrae group (Fig 6B; p = 0.003; Fisher exact test). Thus, similar to our *in vivo* results (Fig 6A, above), absence of sncRNA1&2 significantly upregulated LAT expression in infected Neuro-2A cells.

### Apoptosis is upregulated in Neuro2A cells infected with ΔsncRNA1&2 virus

**Fig 7:**
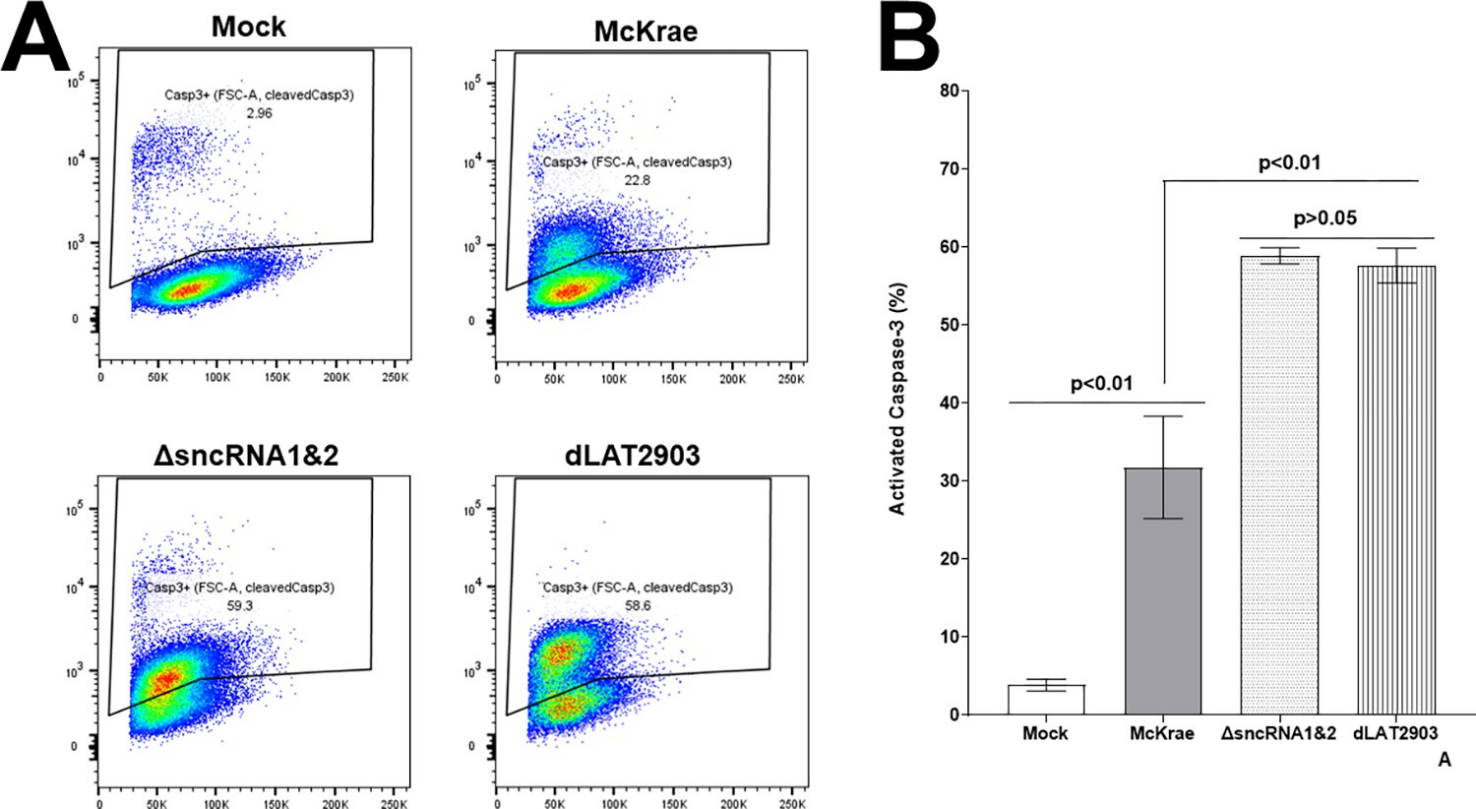
Increase of apoptosis in Neuro-2A cells infected with ΔsncRNA1&2. Neuro-2A cultures were infected with dLAT2903, McKrae, and ΔsncRNA1&2 viruses (10 pfu/cell for 24 hr) or mock infected. (A) Detection of cleaved caspase-3 in infected cells. At 24 hr PI, cells were harvested and reacted with anti-cleaved capase-3 antibody and FACS analysis was performed for expression of caspase-3 (see Materials and Methods). Experiments were repeated twice; and (B) Quantification of FACS analysis from A. Percent of cleaved capase-3 positive cells for each infected or mock infected cells were counted. Each bar represents the mean ± SEM of cleaved capase-3-positive cells (N = 6).

Previously it was shown that HSV-1 can inhibit apoptosis in certain cell types [44]. Later on it was shown that HSV-1 LAT has antiapoptotic function [21,25,45]. In the absence of LAT using dLAT2903 virus, we have shown that levels of apoptosis were higher in dLAT2903 lacking the 2 kb LAT compared with its parental WT strain McKrae [21]. The two sncRNAs are located within the 2 kb LAT and previously it was shown that sncRNA1 and sncRNA2 affects apoptosis in transfected cells *in vitro* [35,36]. Caspase-3 is a main player in apoptosis and can be cleaved by caspase-8 or -9 [46,47]. Thus, to test if levels of apoptosis is affected with the absence of both sncRNA1 and sncRNA2 in ΔsncRNA1&2 compared with dLAT2903 virus, Neuro-2A cells were infected with 10 pfu/cell of McKrae, dLAT2903 and ΔsncRNA1&2 viruses or mock infected. At 24 hr PI, cells were stained with anti-cleaved caspase-3 and the number of caspase-3 positive cells were determined using flow cytometry. Approximately 59.3% of cells infected with ΔsncRNA1&2 were positive for cleaved caspase-3 and this was similar to 58.6% cells in dLAT2903 infected cells and compared with 22.8% in McKrae infected cells (Fig 7A). Only 2.96% of mock infected cells were positive for caspase-3 expression (Fig 7A). Quantitative FACS analyses of infected cells described in Fig 7A revealed significant increases in levels of cleaved caspase-3 between McKrae infected cells compared with ΔsncRNA1&2 and dLAT2903 infected cells (Fig 7B, p< 0.01), while levels of cleaved caspase-3 expressions between ΔsncRNA1&2 and dLAT2903 were similar (Fig 7B, p>0.05). Finally, all infected cells had higher cleaved caspase-3 expression than mock infected cells (Fig 7B, p<0.001). Caspase-3 is a main player in apoptosis [47] and in this study we have shown that enhanced antiapoptotic function of LAT is located within the two sncRNA1 and sncRNA2. Although neuro-2A cells are immortalized but in this study we have shown that LAT expression is similar in RNA isolated from TG of mice infected compared with RNA isolated from infected Neuro-2A cells. These results suggesting that Neuro-2A can be used along with *in vivo* experiments to look at the function of LAT.

## Discussion

HSV-1 is one of the most ubiquitous human pathogens establishing latency in infected host and reactivation of latent virus can cause sight-threatening eye diseases [48–50]. Despite the seriousness of recurrent ocular herpes, no drug or vaccine has been approved by the FDA for prevention of ocular recurrences. Thus, highlighting the critical need for the development of alternative approaches for the prevention and control of HSV-1-induced ocular syndromes. Therefore, discovery of the crucial factor LAT that is responsible for HSV-1 reactivation is necessary to develop therapeutic and prevention strategies for recurrent HSV-1-associated ocular diseases.

Previously Jones’s group has shown that LAT encodes two sncRNAs and are expressed during latency in TG of infected mice [34,36]. In the last decade, sncRNAs has gained fundamental attention due their function as potentially powerful regulators of host and viral gene expression, and their interactions with several key host pathways [51,52]. Thus, sncRNAs became a key part in host-pathogen interactions. Along with RNA viruses, sncRNAs have gained popularity in herpesviruses too and *in vitro* experiments have shown that expression of sncRNA1 and sncRNA2 inhibits productive infection *in vitro* [36]. These two sncRNA1 are expressed during latency in TG of infected mice [34,36]. In addition, sncRNA1 and sncRNA2 have antiapoptotic activity [35,36]. Previously, we reported that the two sncRNAs upregulate HVEM expression *in vitro* [30,37] sncRNA1 has a greater effect on HVEM promoter activity, virus replication and apoptosis than sncRNA2 [35–37]. Mutations of the ATGs within the sncRNA1 or sncRNA2 region significantly reduced HVEM promoter activity. Shen et al., [36] reported that ICP4 protein expression was reduced when ATG was mutated to TTG in transfected cells. They further showed that expression of TTG mutant sncRNA1, but not sncRNA2, restored virus production, suggesting that this ATG is necessary for the sncRNA1 inhibition of viral replication. We have also shown that TTG mutations within the sncRNAs reduced virus replication, apoptosis, and HVEM promoter activity in transfected cells [38]. Thus, some or most of the functions associated with LAT could be mediated by these two sncRNAs.

The two sncRNAs encoded in the HSV-1 LAT locus had been characterized *in vitro*, but their roles *in vivo* have not been studied. Therefore, in order to determine the functions of these two sncRNAs *in vivo*, we constructed a recombinant HSV-1 in McKrae strain lacking sncRNA1 and sncRNA2 regions of LAT (ΔsncRNA1&2). Complete sequence analysis of this recombinant virus has shown that it is similar to WT McKrae except for the missing 62 bp and 36 bp sncRNA1 and sncRNA2 sequences, respectively. *In vitro* virus replication of ΔsncRNA1&2 was similar to that of McKrae and dLAT2903 using RS cells. Similarly, ΔsncRNA1&2 virus replication in the eyes of infected mice was similar to that of McKrae and dLAT2903 viruses and also the mice infected with three viruses had similar levels of eye disease. This is in line with a previous study in which even the absence of the complete 2 kb LAT did not alter virus replication *in vitro* or *in vivo* [16]. Our data also suggests that the absence of both sncRNA1 and sncRNA2 protects the mice mortality in comparison to control viruses as we have shown recently that absence of sncRNA1 leads to more mice mortality [38]. Previously we reported that mice ocularly infected with ΔsncRNA1 virus were more susceptible to ocular infection than mice infected with WT McKrae virus suggesting that the sncRNA1 sequence has a protective role during ocular infection [38]. In contrast, deletion of both sncRNA1&2 in ΔsncRNA1&2 virus did not affect survival in ocularly infected mice compared with WT McKrae or dLAT2903 virus. This is similar to our previous studies showing that the absence of LAT in dLAT2903 did not alter survival in infected mice compared with mice infected with WT McKrae virus [16,20]. Thus, while the absence of sncRNA1 reduces survival, the absence of both sncRNA1&2 in ΔsncRNA1&2 virus have a protective role during ocular infection in mice. Previously we reported that higher mortality rate in ΔsncRNA1 infected mice correlated with decreased *Tnf* expression [38], while the absence of LAT had no effect on *Tnf expression [20]*.

As expected, no differences were detected between ΔsncRNA1&2 and control viruses with regards to primary virus replication *in vitro* or *in vivo*. Original reports on functions of LAT was associated with its role in enhancing latency and increase of virus reactivation in both mice and rabbits [16,17,26]. It has been reported that the antiapoptotic function of LAT contributes to increase of latency-reactivation and conversely LAT-minus viruses have lower levels of latency and also slower reactivation than LAT-plus viruses [16,18,21,40]. The TG of rabbits infected with dLAT2903 virus displayed abundant levels of apoptotic cells compared to the parental wild-type HSV-1 McKrae [21]. LAT was also confirmed for its antiapoptotic function in both human and mouse neuronal cells as well as in additional strains of HSV-1 [25,26,45]. The expression of LAT in mouse neuronal cells were shown to promote inhibition of apoptosis pathways through the regulation of activation of caspase-8 and -9 [53]. Although the precise molecular mechanism involved in antiapoptotic activity remains elusive, earlier reports have shown that LAT may contribute to manipulate and counteract the activation of apoptosis pathways via innate immune signaling pathways [54,55]. Our recent data suggests that one of the mechanisms of inhibition of apoptosis by LAT is downregulation of components of the Type I interferon pathway [27]. Previously it was shown that ATGs mutations within the eight possible LAT ORFs reduces LAT antiapoptotic activity *in vitro* [56]. Thus, antiapoptotic activity appears to be the critical LAT function involved in enhancing the latency-reactivation cycle, because LAT-minus virus can be restored to full wild type reactivation levels by substitution of different antiapoptosis genes (i.e., baculovirus inhibitor of apoptosis protein gene (cpIAP) or cellular FLIP (cellular FLICE-like inhibitory protein) [57–59]. Levels of latency-reactivation as well as apoptosis could be mediated by sncRNA1&2. Similar to HSV-1, bovine herpesvirus 1 (BoHV-1), also encodes at least two sncRNAs within its latency region and they have antiapoptotic activity [35,36,60]. In our current study, in addition to evaluating the latency-reactivation functions of sncRNA1&2, we also looked at antiapoptotic functions of ΔsnRNA1&2 in comparison with that of dLAT2903 (LAT-minus) and WT McKrae (LAT-plus). Caspase-3 is one of crucial and essential mediators of both extrinsic and intrinsic apoptosis pathways [61,62]. Evidence suggests that HSV-1 blocks activation of both caspase-3-independent and dependent apoptosis pathways [44,63]. Caspase-3 has been shown to play critical roles in many different infection models, for instance, in influenza infection that caspase-3 is required for efficient virus progression [64] and in the presence of caspase-3, infection with COVID-19 symptoms can get worse [65].

Our analysis suggest that levels of apoptosis as determined by expressions of cleaved caspase-3 was the same between Neuro2A cells infected with ΔsnRNA1&2 and dLAT2903 viruses and was significantly higher than that of McKrae. These results suggest that similar to the LAT-minus virus (dLAT2903), the antiapoptotic function of LAT is located within the two sncRNAs and that apoptosis reduced in the presence of LAT. Our *in vitro* analysis have shown that antiapoptotic function of LAT is located within the two sncRNAs. Currently we do not know if sncRNA1&2 virus can also increase apoptosis to the level of dLAT2903 *in vivo*. Study is in progress to look at the antiapoptotic functions of the 2 sncRNAs *in vivo* by comparing sncRNA1, sncRNA2, and sncRNA1&2 viruses with one another. We have already reported a recombinant virus lacking sncRNA1 and we are in process of making the sncRNA2 deletion mutant virus. After generation of sncRNA2, our plan is to compare the three viruses with one another both *in vitro* and *in vivo* to determine the contribution of each of the sncRNAs to apoptosis and if the effect is additive or one contribute to apoptosis, while the other does not. This study also shows that higher apoptosis is independent of virus reactivation since the time to reactivation remains the same between McKrae and ΔsnRNA1&2 viruses. In dLAT2903 (LAT-minus) virus, levels of viral DNA (gB copy number) were lower than McKrae (LAT-plus) virus but in the ΔsnRNA1&2 virus, gB expression levels were intermediate to that of McKrae and dLAT2903 viruses. Furthermore, LAT expression levels were seen to be higher in the absence of snRNA1&2 regions both *in vitro* and *in vivo* in comparison to McKrae virus. Even though presence of LAT influences apoptosis, levels of latency and time to reactivation but in this study, we have shown that higher expression of LAT both *in vitro* and *in vivo* did not affect levels of apoptosis or virus reactivation. Although, while average of reactivation time between ΔsnRNA1&2 virus with McKrae virus was not significantly different but there was a tendency of tighter reactivation time in ΔsnRNA1&2 infected TG than McKrae virus. Thus, our results suggest that increase in the levels of apoptosis in the absence of LAT over that of LAT-plus viruses maybe located within the two sncRNAs and is independent of expression levels of viral DNA, LAT, and time to reactivation.

In summary, many functions have been associated with the 2 kb LAT and these multiple functions of LAT has been studied using recombinant viruses that lack LAT or contain truncated versions of LAT that could perhaps also disrupt individual sncRNAs. In this study by deleting the two sncRNAs within the LAT stable 2 kb, we have separated enhanced antiapoptotic functions of LAT from other LAT functions. To recapitulate, our results of this study suggest a protective role for sncRNA1&2 during HSV-1 ocular infection and that the enhanced function of HSV-1 LAT is encoded within the two sncRNAs.

## Materials and methods

### Ethics statement

All animal procedures were performed in strict accordance with the Association for Research in Vision and Ophthalmology Statement for the Use of Animals in Ophthalmic and Vision Research and the NIH *Guide for the Care and Use of Laboratory Animals* (ISBN 0-309-05377-3). Animal research protocols were approved by the Institutional Animal Care and Use Committee of Cedars-Sinai Medical Center (Protocols #5030 and #8837).

### Viruses, cells, and mice

Plaque-purified HSV-1 strains McKrae (wild type, LAT-plus), dLAT2903 (LAT-minus in which both copies of the LAT promoter (one in each viral long repeat) and the first 1,667 nucleotides (nt) of the LAT transcript are deleted), and ΔsncRNA1&2 described below that both its sncRNA1 and 2 were deleted in both long repeat were grown in rabbit skin (RS) cell monolayers in minimal essential medium (MEM) containing 5% fetal bovine serum (FBS), as described previously [16,66]. Neuro-2A cells (CCL 131, American Type Culture Collection) were maintained in Dulbecco’s modified Eagle medium (DMEM) with 10% FBS. The Neuro-2A cells are used as a representative tissue culture of neuronal type cell for studying LAT’s effect on apoptosis [21,25]. Wild-type C57BL/6 mice were purchased from Jackson Laboratories.

### Construction of sncRNA1&2 deleted plasmid

The parental virus for this construct was dLAT2903, a recombinant of HSV-1 strain McKrae in which the region of LAT from -161 to +1667 relative to the LAT transcription start site (an EcoRV-HpaI restriction enzyme fragment) was deleted from both copies of LAT [16]. Thus, this LAT-null recombinant is missing approximately 0.2 kb of the LAT promoter and 1.6 kb of the 5’ end of the primary 8.3-kb LAT transcript. To make ΔsncRNA1&2 virus, the BamHI B fragment of McKrae was digested with SwaI and BamHI to produce a 5.5-Kb DNA fragment including –1041 to +4656 of the HSV-1 LAT [67]. A PacI linker was added to the 5.5-Kb fragment and ligated into the PacI site of modified pNEB193 (New England Biolab) lacking its internal BamHI site. The resulting plasmid was digested with StyI-HpaI to remove the 1.6-Kb LAT fragment corresponding to LAT +76 to +1667 nt. A BamHI linker was added, and the resulting plasmid was designated pLAT [67]. pLAT contained 880 bp upstream, and 2989 bp downstream, of the BamHI site. A plasmid containing the region of LAT from -161 to +1667 relative to the LAT transcription start site and without the sncRNA1&2 regions relative to whole HSV-1 genome (119850–119912) was synthesized (GenScript, Piscataway, NJ) and inserted into the BamH1 site of pLAT as we described previously [67]. The resulting plasmid was designated pLAT-ΔsncRNA1&2.

### Generation and confirmation of ΔsncRNA1&2 virus

ΔsncRNA1&2 virus was generated by homologous recombination as we previously described [16,67]. Briefly, pLAT-ΔsncRNA1&2 was co-transfected with infectious dLAT2903 viral DNA by the calcium phosphate method. Viruses from the co-transfection were plated, and isolated plaques were picked and screened for LAT insertion using restriction digestion. Plaques containing LAT were selected, plaque purified five times, and reanalyzed by restriction digestion to ensure that LAT was present in the LAT region of dLAT2903 virus. A final plaque was purified and designated ΔsncRNA1&2. Genomic DNA was phenol chloroform extracted from ΔsncRNA1&2 and WT HSV-1 McKrae, and DNA library preparations, sequencing reactions, and bioinformatics analysis were conducted at GENEWIZ, LLC. (South Plainfield, NJ, USA). Genomic DNA was quantified using the Qubit 2.0 Fluorometer (Life Technologies, Carlsbad, CA, USA). DNA integrity was checked with 0.6% agarose gel with 50–60 ng sample loaded in the well. RNase A from QIAGEN or RNase If from NEB was used to perform RNase Treatment for the samples that had RNA contamination prior to performing library preparation. Subsequently, NEBNext Ultra II DNA Library Prep Kit for Illumina, clustering, and sequencing reagents was used throughout the process following the manufacturer’s recommendations. Briefly, the genomic DNA was fragmented by acoustic shearing with a Covaris S220 instrument. Fragmented DNA was cleaned up and end repaired. Adapters were ligated after adenylation of the 3’ends followed by enrichment by limited cycle PCR. DNA libraries were validated using a DNA 1000 Chip on the Agilent 2100 Bioanalyzer (Agilent Technologies, Palo Alto, CA, USA), and were quantified using Qubit 2.0 Fluorometer. The DNA libraries were also quantified by real time PCR (Applied Biosystems, Carlsbad, CA, USA), clustered on 1 lane of a flowcell, and loaded on the Illumina MiSeq instrument according to manufacturer’s instructions. The samples were sequenced using a 2x150 paired end (PE) configuration. Sequencing was performed using a 2x250 PE configuration; image analysis and base calling was conducted by the MiSeq Control Software (MCS) on the MiSeq instrument. Sequence reads for each sample were trimmed for their adapter sequences and nucleotides with poor quality using Trimmomatic. After trimming reads shorter than 35 bases were discarded. The reads were then mapped to the published genome of McKrae MN136524 [68] using the Burrows-Wheeler Alignment Tool (BWA, v.0.7.12.). From a total of 76,323,123 reads, 20,475,451 reads were mapped. The bam files from BWA alignment were sorted. Consensus and summary of mapped reads at each position are extracted from the formatted bam file using Samtools mpileup. Variants are then called from the mpileup output using VarScan with the following settings: minimum coverage is 10, minimum number of reads 2 is 7, minimum variant frequency is 25% and highest p-value is 0.05. After the alignment completed, structural variations (SV) including large INDELs were detected using the program, Manta. However, no structural variant was detected from these samples. Sequences were analyzed using Integrative Genomics Viewer version 2.8.10 (IGV, Broad institute, CA) and the complete sequences of the recombinant ΔsncRNA1&2 virus is shown in S1 Fig. The complete genome sequences for ΔsncRNA1&2 virus and WT McKrae virus have been deposited at GenBank under the accession numbers OR723971 and OL638991, respectively.

### Determination of virus kinetics *in vitro*

RS cell monolayers were infected with 0.1, 1, and 10 pfu/cell of WT HSV-1 strain McKrae, dLAT2903, or ΔsncRNA1&2 virus for 12, 24, or 48 hr, after which plates were frozen at -80°C. Virus titers were determined by standard plaque assays in RS cells.

### Ocular infection and virus titers in the eyes of infected mice

Mice were infected ocularly with 2 x 10^5^ pfu of each virus. Each virus was suspended in 2 μl of tissue culture media and administered as an eye drop without prior corneal scarification. Tears were collected on days 1–7 PI using cotton swabs [69]. Individual swabs were placed in 1 ml tissue culture media. Viral titers were determined using standard plaque assays in RS cells.

### Monitoring survival, corneal scarring, and angiogenesis

Mice survival was monitored for 28 days and the presence of eye disease in surviving mice were assessed in a blinded fashion. CS was scored on a scale of 0 to 4 (0 = no disease, 1 = mild hazing, 2 = moderate opacity, 3 = severe corneal opacity, but iris visible, 4 = opaque and corneal ulceration). Angiogenesis was scored by assessing the presence of new vessel growth in each of the four corneal quadrants on a scale of 0–16. Scores of each quadrant were summed and mean ± SEM are shown [70].

### *Ex-vivo* explant reactivation assay

Individual TG were removed from mice on day 28 PI, placed in tissue culture medium, and cultured in a humidified 37°C incubator supplemented with 5% CO_2_ [71,72]. Aliquots of culture medium from the explants were transferred onto indicator RS cells daily for 10 days and the cells were monitored for cytopathic effect. Media from explanted TG cultures was plated daily, and the time at which reactivated virus first appeared in explanted TG cultures was determined.

### DNA extraction and PCR analysis for HSV-1 genomic DNA

DNA was isolated from homogenized individual TG using the commercially available Dneasy Blood &Tissue Kit (Qiagen, Stanford, CA Cat.No. 69506) according to the manufacturer’s instructions. PCR analyses were done using gB specific primers (Forward—5’-AACGCGACGCACATCAAG-3’; Reverse—5’-CTGGTACGCGATCAGAAAGC-3’; and Probe—5’-FAM-CAGCCGCAGTACTACC-3’). The amplicon Length for this primer set is 72 bp. Relative copy numbers for gB DNA were calculated using standard curves generated from the plasmid pAc-gB1. In all experiments, GAPDH was used for normalization of all measured transcripts.

### Apoptosis in Neuro-2A infected cells

Neuro-2A cells can be infected efficiently with HSV-1 but virus replication in infected cells are not as high as RS cells. Cells were grown to 70–80% confluency and infected with 10 pfu/cell of McKrae, dLAT2903 and ΔsncRNA1&2 viruses or mock infected. At 24 hr PI, cells were harvested, washed, resuspended in FACS buffer and incubated for 15 min at 4°C with purified 2.4G2 antibody (Fc block, BD Biosciences, San Diego, CA) followed by subsequent incubation with Alexa Fluor 647 cleaved caspase-3 Rabbit mAb {Asp175 (D3E9)}, Cell Signaling Technology, Danvers, MA) at 4°C for 1 hr followed by fixation with BD Cytofix/Cytoperm solution for 20 min at 4°C. The cells were washed again and analyzed using FACScan instrumentation (Becton Dickinson). Experiment were performed twice (n = 6).

### LAT expression in Neuro-2A infected cells

Neuro-2A cells were grown to confluency as above and infected with 0.1 pfu/cell of McKrae or ΔsncRNA1&2 virus for 12, 24 and 48 hr. At 12, 24, and 48 hr PI, cells were harvested, washed, and total RNA was isolated and RT-PCR on isolated RNA was performed as described below.

### RNA extraction, cDNA synthesis, and TaqMan RT-PCR

TG from individual mice were collected on day 28 PI, immersed in RNA stabilization reagent (RNA Later, Thermo Fisher Scientific, Waltham, MA, USA), and stored at −80°C until processing. Total RNA was extracted as described [30,73]. Primer probe sets consisted of two unlabeled PCR primers and the FAM dye-labeled TaqMan MGB probe formulated into a single mixture. Additionally, all cellular amplicons included an intron-exon junction to eliminate signal from genomic DNA contamination. Levels of LAT RNA from latent TG were determined using custom-made LAT primers and probe as follows: forward primer, 5′-GGGTGGGCTCGTGTTACAG-3′; reverse primer, 5′-GGACGGGTAAGTAACAGAGTCT CTA-3′; and probe, 5′-FAM-ACACCAGCCCGTTCTTT-3′ (amplicon length = 81 bp). Copy number of LAT RNA was calculated using a standard curve generated using pGEM5317-LAT as we described previously [71].

GAPDH was used as a loading control in all experiments. Quantitative real-time PCR (qRT-PCR) was performed using the TaqMan gene expression assay kit in 384-well plates on ABI QuantStudio 5 (Applied Biosystems, Foster City, CA). Real-time PCR was performed in triplicate for each tissue sample. The threshold cycle (C_t_) values, representing the PCR cycle at which there is a noticeable increase in reporter fluorescence above baseline, were determined using SDS 2.2 software.

### Statistical analysis

Student’s t tests and Tukey’s multiple comparison tests were performed using the computer program Instat (GraphPad, San Diego, CA). Results were considered statistically significant when the "P" value was <0.05.

## Supporting information

S1 Fig Complete genome sequencing of ΔsncRNA1&2 recombinant virus. Whole genome sequencing of ΔsncRNA1&2 virus confirmed absence of (A and B) sncRNA1 sequence (5’-GCCTGTGTTTTTGTGCCTGGCTCTCTATGCTTGGGTCTTACTGCCTGGGGGGGGGGAGTGCG-3’) at **(A)** position 119,887–199,948 on positive strand and **(B)** 6,476–6,537 on negative strand, and absence of sncRNA2 sequence (5’-CATTCTTGTTTTCTAACTATGTTCCTGTTTCT GTCT-3) at position **(C)** 120,280–120,315 on positive strand and **(D)** 6,109–6,144 on negative strand.
(PDF)

S1 Data Data that underlies this paper. (PDF)
