# Supplementary material for: The anti-apoptotic function of HSV-1 LAT in neuronal cell cultures but not its function during reactivation correlates with expression of two small non-coding RNAs, sncRNA1&2
Source: PLoS Pathog. 2024 Jun 10;20(6):e1012307. doi: 10.1371/journal.ppat.1012307 (PMC11192303; doi:10.1371/journal.ppat.1012307)
Supplement: S1 Fig — Whole genome sequencing of ΔsncRNA1&2 virus confirmed absence of (A and B) sncRNA1 sequence (5’-GCCTGTGTTTTTGTGCCTGGCTCTCTATGCTTGGGTCTTACTGCCTGGGGGGGGGGAGTGCG-3’) at (A) position 119,887–199,948 on positive strand and (B) 6,476–6,537 on negative strand, and absence of sncRNA2 sequence (5’-CATTCTTGTTTTCTAACTATGTTCCTGTTTCT GTCT-3) at position (C) 120,280–120,315 on positive strand and (D) 6,109–6,144 on negative strand. (PDF) [file ppat.1012307.s001.pdf]

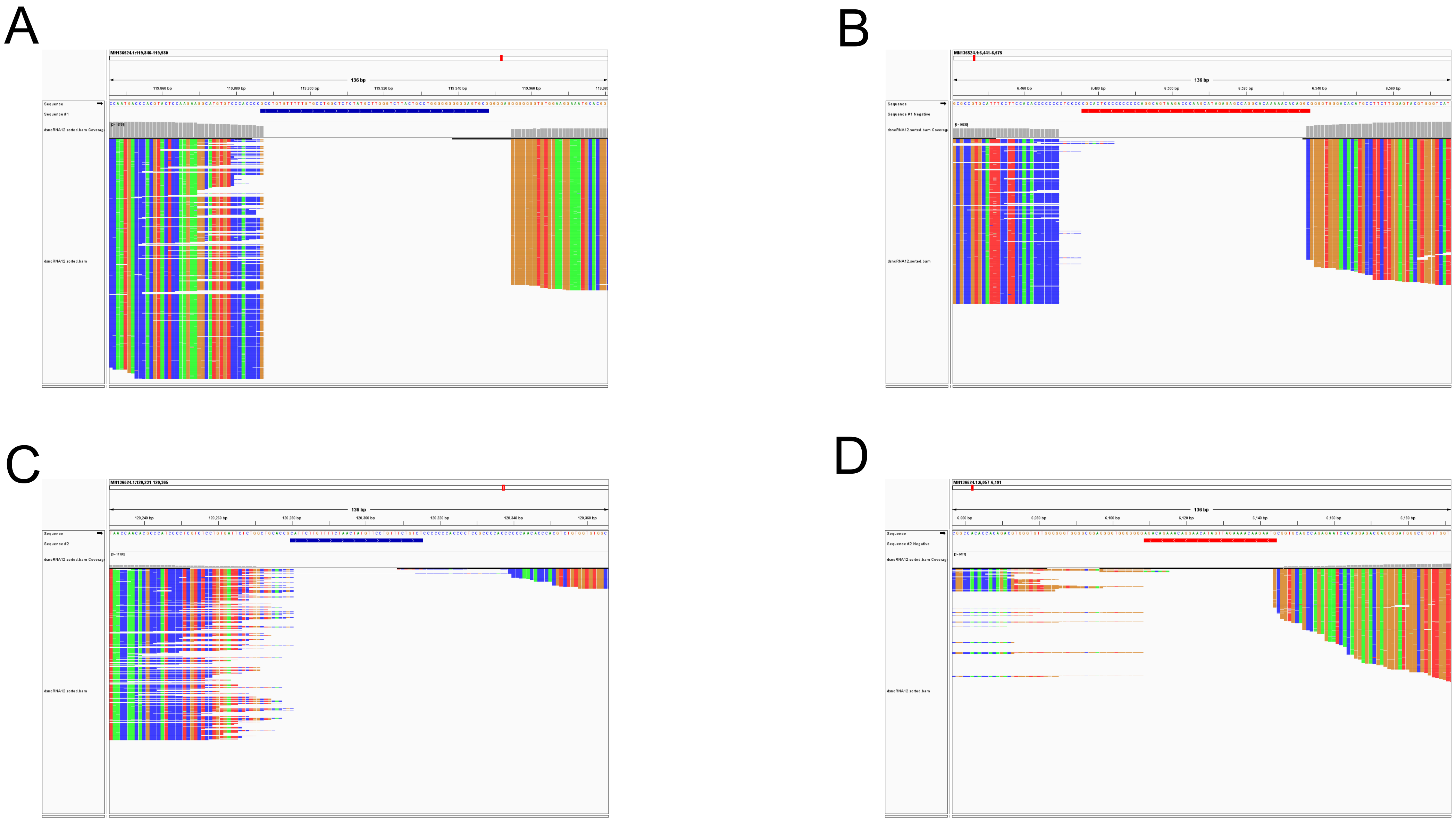

<u>Fig. S1. Complete genome sequencing of ΔsncRNA1&2 recombinant virus</u>. Whole genome sequencing of ΔsncRNA1&2 virus confirmed absence of (A and B) sncRNA1 sequence (5’-GCCTGTGTTTTTGTGCCTGGCTCTCTATGCTTGGGTCTTACTGCCTGGGGGGGGGGAGTGCG-3’) at **(A)** position 119,887 – 199,948 on positive strand and **(B)** 6,476 – 6,537 on negative strand, and absence of sncRNA2 sequence (5’-CATTCTTGTTTTCTAACTATGTTCCTGTTTCTGTCT-3) at position **(C)** 120,280 – 120,315 on positive strand and **(D)** 6,109 – 6,144 on negative strand.
