# Supplementary material for: The anti-apoptotic function of HSV-1 LAT in neuronal cell cultures but not its function during reactivation correlates with expression of two small non-coding RNAs, sncRNA1&2
Source: PLoS Pathog. 2024 Jun 10;20(6):e1012307. doi: 10.1371/journal.ppat.1012307 (PMC11192303; doi:10.1371/journal.ppat.1012307)
Supplement: S1 Data — (PDF) [file ppat.1012307.s002.pdf]

Table format: **XY**		X	Group A			Group B			Group C		
		X Title	sncRNA1&2			McKrae			dLAT2903		
		X	A:Y1	A:Y2	A:Y3	B:Y1	B:Y2	B:Y3	C:Y1	C:Y2	C:Y3
1	12		2500	1633	2417	500	567	500	1633	1500	1183
2	24		1275000	650000	1000000	2125000	1400000	1550000	550000	600000	675000
3	48		5250000	5500000	2750000	4750000	2250000	4250000	1750000	2250000	3000000

Data for Figure 2A_Measuring Virus Yield in vitro 0.1 PFU/cell

Table format: **Grouped**		Group A sncRNA1&2			Group B McKrae			Group C dLAT29032903		
		A:1	A:2	A:3	B:1	B:2	B:3	C:1	C:2	C:3
1	12	87500	22500	45000	125000	62500	120000	20000	22500	57500
2	24	6250000	4500000	2250000	4500000	6250000	5250000	2750000	3000000	2250000
3	48	8500000	4250000	5750000	6250000	9000000	4750000	5000000	5000000	5000000

Data for Figure 2B_Measuring virus yield in vitro_1.0 PFU/Cell

Table format: **Grouped**		Group A			Group B			Group C		
		sncRNA1&2			McKrae			dLAT2903		
		A:1	A:2	A:3	B:1	B:2	B:3	C:1	C:2	C:3
1	12	500000.000	475000.000	375000.000	675000.000	525000.000	825000.000	450000.0	350000.0	450000.0
2	24	1.750e+007	1.750e+007	1.500e+007	7250000.000	5000000.000	3250000.000	6500000.0	3750000.0	5750000.0
3	48	2.625e+007	2.075e+007	2.075e+007	2.125e+007	2.150e+007	1.875e+007	2.6e+007	1.7e+007	1.8e+007

Data for Figure 2C_Measuring virus yield in vitro 10 PFU/cell

Table format: XY		X										Grou
		Days Post infection										Mck
		X	A:Y1	A:Y2	A:Y3	A:Y4	A:Y5	A:Y6	A:Y7	A:Y8	A:Y9	A:Y10
1	Title	1	940	1500	3560	690	2400	1840	1840	2480	2280	2160
2	Title	2	48000	29800	400	12600	51800	1600	800	6200	4600	*200**
3	Title	3	1400	17000	400	200	*0**	*0**	600	800	600	400
4	Title	4	4800	*0**	9200	4400	2000	5600	2000	3000	*600**	5000
5	Title	5	2000	150	950	470	1650	920	70	170	2060	3560
6	Title	6	0	90	190	0	0	0	120	20	0	0
7	Title	7	0	0	0	0	0	0	0	30	0	0

Data for Figure 3_Eye Swab Virus Titer

	up A										
	Krae										
	A:Y11	A:Y12	A:Y13	A:Y14	A:Y15	A:Y16	A:Y17	A:Y18	A:Y19	A:Y20	B:Y1	B:Y2
1	850	1120	450	210	3680	290	450	450	3560	850	1750	50
2	27600	14800	35400	600	46400	2400	600	600	41600	2000	400	4400
3	1200	2600	1200	11200	6400	200	5000	600	200	*0**	40000	6000
4	24800	5600	5800	2600	1200	4800	1600	4600	4400	1000	800	3200
5	790	450	900	100	2760	2400	2360	1860	1010	1230	30	680
6	0	10	0	30	70	20	20	60	20	110	0	400
7	0	0	0	0	0	0	40	0			0	60

Data for Figure 3_Eye Swab Virus Titer

	Group B											
	ΔsncRNA1&2											
	B:Y3	B:Y4	B:Y5	B:Y6	B:Y7	B:Y8	B:Y9	B:Y10	B:Y11	B:Y12	B:Y13	B:Y14
1	30	0	20	110	730	190	790	110	1590	90	10	330
2	9400	3000	17800	12800	4000	*0**	7200	2200	3400	3000	11200	4400
3	200	600	200	0	0	600	3000	8000	1400	16800	600	*0**
4	600	4600	400	600	3000	0	33600	2000	8000	0	2000	400
5	0	920	30	1790	20	1160	300	0	320	450	1600	4000
6	0	0	0	0	200	0	0	0	0	0	200	0
7	0	0	120	0	0	120	10	0	0	0	0	0

Data for Figure 3_Eye Swab Virus Titer

	B:Y15	B:Y16	B:Y17	B:Y18	B:Y19	B:Y20	C:Y1	C:Y2	C:Y3	C:Y4	C:Y5	C:Y6
1	350	880	1090	200	0	0	1560	3560	350	90	1740	80
2	9200	5200	*0**	12400	4200	*0**	14200	800	4400	1000	21600	8600
3	2400	1000	800	1800	200	*0**	5200	*0**	*0**	2600	6400	3400
4	0	2800	11000	1400	0	5200	4600	2400	7200	2000	6400	9600
5	0	3000	2400	1700	480	480	200	400	800	600	200	600
6	0	0	0	0	0	0	600	0	0	0	600	0
7	0	0	0	230			0	430	0	0	0	0

Data for Figure 3_Eye Swab Virus Titer

	Group C											
	dLAT2903											
	C:Y7	C:Y8	C:Y9	C:Y10	C:Y11	C:Y12	C:Y13	C:Y14	C:Y15	C:Y16	C:Y17	C:Y18
1	220	90	640	1060	290	1720	2020	300	1640	110	90	1050
2	5000	7000	13200	5800	3000	18800	7000	12200	4000	15800	13400	9600
3	400	800	9400	1200	3200	2400	11400	7600	12800	14400	5000	5800
4	2800	1200	2000	1800	1600	6000	1000	5000	18800	2400	32000	*800**
5	600	4000	2400	1200	12000	1000	3000	0	4200	4600	12800	2800
6	0	0	0	0	800	0	0	0	0	0	0	0
7	0	150	0	10	0	0	0	0	180	0		

Data for Figure 3_Eye Swab Virus Titer

	C:Y19	C:Y20
1	70	2640
2	9600	17400
3	*0**	1600
4	2600	6600
5	2600	1600
6	0	0
7		

Data for Figure 3_Eye Swab Virus Titer

Data for Figure 4A_Survival

Table format: **XY**		X	Group A	Group B	Group C
		Days Post Infection	McKrae	ΔsncRNA1&2	dLAT2903
		X	Y	Y	Y
1	Title	7	1	1	1
2	Title	7			1
3	Title	8			
4	Title	8			
5	Title	9	1	1	1
6	Title	9		1	1
7	Title	9		1	
8	Title	10			
9	Title	10			
10	Title	11			
11	Title	12			
12	Title	13			
13	Title	14	1		
14	Title	14	0	0	0
15	Title	14	0	0	0
16	Title	15	0	0	0
17	Title	15	0	0	0
18	Title	15	0	0	0
19	Title	15	0	0	0
20	Title	28	0	0	0
21	Title	28	0	0	0
22	Title	28	0	0	0
23	Title	28	0	0	0
24	Title	7	1	1	
25	Title	8			
26	Title	8			
27	Title	8			
28	Title	9	1		
29	Title	9	1		
30	Title	9			
31	Title	10	1		

Table format: **XY**		X	Group A	Group B	Group C
		Days Post Infection	McKrae	ΔsncRNA1&2	dLAT2903
		X	Y	Y	Y
32	Title	10			
33	Title	11			
34	Title	12			
35	Title	13	1		
36	Title	13			
37	Title	14	0	0	0
38	Title	14	0	0	0
39	Title	15	0	0	0
40	Title	15	0	0	0
41	Title	15	0	0	0
42	Title	15	0	0	0
43	Title	28	0	0	0
44	Title	28	0	0	0
45	Title	28	0	0	0
46	Title	28	0	0	0
47	Title	9	1	1	1
48	Title	9	1	1	1
49	Title	9			
50	Title	9			
51	Title	9			
52	Title	9			
53	Title	9			
54	Title	10			1
55	Title	10			
56	Title	11			
57	Title	12			
58	Title	13			
59	Title	13			
60	Title	14	0	0	0
61	Title	14	0	0	0
62	Title	15	0	0	0

Data for Figure 4_Survival

Table format: **XY**		X	Group A	Group B	Group C
		Days Post Infection	McKrae	ΔsncRNA1&2	dLAT2903
		X	Y	Y	Y
63	Title	15	0	0	0
64	Title	15	0	0	0
65	Title	15	0	0	0
66	Title	28	0	0	0
67	Title	28	0	0	0
68	Title	28	0	0	0
69	Title	28	0	0	0
70	Title	9		1	
71	Title	9		1	
72	Title	9		1	
73	Title	9			
74	Title	9			
75	Title	9			
76	Title	9			
77	Title	10			
78	Title	10			
79	Title	11	1		
80	Title	11	0	0	0
81	Title	11	0	0	0
82	Title	11	0	0	0
83	Title	12			
84	Title	13			
85	Title	13			
86	Title	14	0	0	0
87	Title	14	0	0	0
88	Title	15	0	0	0
89	Title	15	0	0	0
90	Title	15	0	0	0
91	Title	15	0	0	0
92	Title	28	0	0	0
93	Title	28	0	0	0

Data for Figure 4A_Survival

Data for Figure 4A_Survival

Table format: XY		X	Group A	Group B	Group C
		Days Post Infection	McKrae	ΔsncRNA1&2	dLAT2903
		X	Y	Y	Y
94	Title	28	0	0	0
95	Title	28	0	0	0
96	Title	8	1	1	
97	Title	8		1	
98	Title	8			
99	Title	9			
100	Title	9			
101	Title	9			
102	Title	10			
103	Title	10			
104	Title	11			
105	Title	12			
106	Title	13			
107	Title	13			
108	Title	14	0	0	0
109	Title	14	0	0	0
110	Title	15	0	0	0
111	Title	15	0	0	0
112	Title	15	0	0	0
113	Title	15	0	0	0
114	Title	28	0	0	0
115	Title	28	0	0	0
116	Title	28	0	0	0
117	Title	28	0	0	0

Table format: **XY**		X	
		X Title	

		X	A:Y1	A:Y2	A:Y3	A:Y4	A:Y5	A:Y6	A:Y7	A:Y8	A:Y9	A:Y10	A:Y11
1	Title		2.0	3	3.5	3	0	4	1	*0.5**	5	2	4.5

Data for Figure 4B_Corneal Scarring

	A:Y12	A:Y13	A:Y14	A:Y15	A:Y16	A:Y17	A:Y18	A:Y19	A:Y20	A:Y21	A:Y22	A:Y23
1	5	1	*0.5**	3	4	1	1	1	3.5	4	3.5	1

Data for Figure 4B_Corneal Scarring

	A:Y24	A:Y25	A:Y26	A:Y27	A:Y28	A:Y29	A:Y30	A:Y31	A:Y32	A:Y33	A:Y34	A:Y35
1	1	1	1	1	0.5	4	2	4	2.5	0.5	0.5	4

Data for Figure 4B_Corneal Scarring

	A:Y36	A:Y37	A:Y38	A:Y39	A:Y40	A:Y41	A:Y42	A:Y43	A:Y44	A:Y45	A:Y46	A:Y47
1	5	2	4	1	2.5	3.5	5	1	1.5	4	1	1.5

Data for Figure 4B_Corneal Scarring

	A:Y48	A:Y49	A:Y50	A:Y51	A:Y52	A:Y53	A:Y54	A:Y55	A:Y56	A:Y57	A:Y58	A:Y59
1	4	1.5	1	3	4	1	0.5	1.5	0.5	0.5	1	4

Data for Figure 4B Corneal Scarring

	A:Y60	A:Y61	A:Y62	A:Y63	A:Y64	A:Y65	A:Y66	A:Y67	A:Y68	A:Y69	A:Y70	A:Y71
1	1	1	4	1.5	4	3	4	1.5	2	4	4	0.5

Data for Figure 4B Corneal Scarring

	Group A											
	McKrae											
	A:Y72	A:Y73	A:Y74	A:Y75	A:Y76	A:Y77	A:Y78	A:Y79	A:Y80	A:Y81	A:Y82	A:Y83
1	0	0	0.0	0	3.5	0.0	4.0	0.0	4.0	2.0	0.5	0.0

Data for Figure 4B Corneal Scarring

	A:Y84	A:Y85	A:Y86	A:Y87	A:Y88	A:Y89	A:Y90	A:Y91	A:Y92	A:Y93	A:Y94	A:Y95
1	2.0	0.75	0.0	1.0	0.0	4.0	0.0	0.0	0.0	0.0	0.0	3.5

Data for Figure 4B Corneal Scarring

	A:Y96	A:Y97	A:Y98	A:Y99	A:Y100	A:Y101	A:Y102	A:Y103	A:Y104	A:Y105	A:Y106	A:Y107
1	0.0	0.0	2.0	2.0	0.0	2.0	4.0	4.0	2.0	0.5	0.00	3.0

Data for Figure 4B Corneal Scarring

	A:Y108	A:Y109	A:Y110	A:Y111	A:Y112	A:Y113	A:Y114	A:Y115	A:Y116	A:Y117	A:Y118	A:Y119
1	0.0	0.0	0.75	3.5	1.0	0.5	3.0	4.0	1.0	2.5	0.0	3.0

Data for Figure 4B Corneal Scarring

	A:Y120	A:Y121	A:Y122	A:Y123	A:Y124	A:Y125	A:Y126	A:Y127	A:Y128	A:Y129	A:Y130	A:Y131
1	3.0	0.0	0.0	4.0	2.0	4.0	1.5	2.0	0.0			

Data for Figure 4B Corneal Scarring

	A:Y132	A:Y133	A:Y134	A:Y135	A:Y136	A:Y137	A:Y138	A:Y139	A:Y140	A:Y141	A:Y142	A:Y143
1												

Data for Figure 4B Corneal Scarring

	B:Y1	B:Y2	B:Y3	B:Y4	B:Y5	B:Y6	B:Y7	B:Y8	B:Y9	B:Y10	B:Y11	B:Y12
1	*0**	4	4	4.5	4	3	1	4.0	4.5	4.5	4	1

Data for Figure 4B Corneal Scarring

	B:Y13	B:Y14	B:Y15	B:Y16	B:Y17	B:Y18	B:Y19	B:Y20	B:Y21	B:Y22	B:Y23	B:Y24
1	0.5	0.5	0.5	1.5	1	0.5	0.5	2.5	0.5	0.5	0.5	1.5

Data for Figure 4B Corneal Scarring

	B:Y25	B:Y26	B:Y27	B:Y28	B:Y29	B:Y30	B:Y31	B:Y32	B:Y33	B:Y34	B:Y35	B:Y36
1	1	1.5	1	0.5	5	2	1	2.5	0	0.5	0.5	2

Data for Figure 4B_Corneal Scarring

	B:Y37	B:Y38	B:Y39	B:Y40	B:Y41	B:Y42	B:Y43	B:Y44	B:Y45	B:Y46	B:Y47	B:Y48
1	0.5	1	0.5	0.5	4	3.5	3.5	4	3	4	0.5	0.5

Data for Figure 4B Corneal Scarring

	B:Y49	B:Y50	B:Y51	B:Y52	B:Y53	B:Y54	B:Y55	B:Y56	B:Y57	B:Y58	B:Y59	B:Y60
1	3.5	4	4	4	2.5	1	4	4.5	0	0.5	1	1.5

Data for Figure 4B Corneal Scarring

	Group B											
	ΔsncRNA1&2											
	B:Y61	B:Y62	B:Y63	B:Y64	B:Y65	B:Y66	B:Y67	B:Y68	B:Y69	B:Y70	B:Y71	B:Y72
1	1	0.5	1.5	4	0.5	0.5	1	1	1.5	3	1	4

Data for Figure 4B Corneal Scarring

	B:Y73	B:Y74	B:Y75	B:Y76	B:Y77	B:Y78	B:Y79	B:Y80	B:Y81	B:Y82	B:Y83	B:Y84
1	0.5	4	1	4	0.5	0	4	2.5	0	4	0	0

Data for Figure 4B Corneal Scarring

	B:Y85	B:Y86	B:Y87	B:Y88	B:Y89	B:Y90	B:Y91	B:Y92	B:Y93	B:Y94	B:Y95	B:Y96
1	3	4	0	4	2	0.5	3.5	4	0	1	0	3

Data for Figure 4B Corneal Scarring

	B:Y97	B:Y98	B:Y99	B:Y100	B:Y101	B:Y102	B:Y103	B:Y104	B:Y105	B:Y106	B:Y107	B:Y108
1	0	4	4	0.5	4	4	2.5	0.5	0	2	4	4

Data for Figure 4B Corneal Scarring

	B:Y109	B:Y110	B:Y111	B:Y112	B:Y113	B:Y114	B:Y115	B:Y116	B:Y117	B:Y118	B:Y119	B:Y120
1	2	0	0	0	1	1.5	3	2.5	3.5	3	1	0.75

Data for Figure 4B Corneal Scarring

	B:Y121	B:Y122	B:Y123	B:Y124	B:Y125	B:Y126	B:Y127	B:Y128	B:Y129	B:Y130	B:Y131	B:Y132
1	3.5	4	0	4	4	1	0	4				

Data for Figure 4B Corneal Scarring

	B:Y133	B:Y134	B:Y135	B:Y136	B:Y137	B:Y138	B:Y139	B:Y140	B:Y141	B:Y142	B:Y143	C:Y1
1												1

Data for Figure 4B Corneal Scarring

	C:Y2	C:Y3	C:Y4	C:Y5	C:Y6	C:Y7	C:Y8	C:Y9	C:Y10	C:Y11	C:Y12	C:Y13
1	4	5	4	1	5	4	3.5	4	4	1	4	0.5

Data for Figure 4B Corneal Scarring

	C:Y14	C:Y15	C:Y16	C:Y17	C:Y18	C:Y19	C:Y20	C:Y21	C:Y22	C:Y23	C:Y24	C:Y25
1	0.5	0	0.5	0.5	1.5	0.5	4.5	0.5	3.5	4	1.5	4

Data for Figure 4B Corneal Scarring

	C:Y26	C:Y27	C:Y28	C:Y29	C:Y30	C:Y31	C:Y32	C:Y33	C:Y34	C:Y35	C:Y36	C:Y37
1	4	0.5	4	3	3	1	3	1	4	0.5	1	0

Data for Figure 4B Corneal Scarring

	C:Y38	C:Y39	C:Y40	C:Y41	C:Y42	C:Y43	C:Y44	C:Y45	C:Y46	C:Y47	C:Y48	C:Y49
1	4.5	4	2	1	4	4	4	4	4	2	2.5	2.5

Data for Figure 4B Corneal Scarring

	C:Y50	C:Y51	C:Y52	C:Y53	C:Y54	C:Y55	C:Y56	C:Y57	C:Y58	C:Y59	C:Y60	C:Y61
1	1.5	4	1.5	1	0.5	2	1	0.5	0.5	1	2.5	1

Data for Figure 4B Corneal Scarring

												Group C
												dLAT2903
	C:Y62	C:Y63	C:Y64	C:Y65	C:Y66	C:Y67	C:Y68	C:Y69	C:Y70	C:Y71	C:Y72	C:Y73
1	1	1.5	0.5	1	0.5	3	0.5	1	1	1	0.5	1

Data for Figure 4B Corneal Scarring

	C:Y74	C:Y75	C:Y76	C:Y77	C:Y78	C:Y79	C:Y80	C:Y81	C:Y82	C:Y83	C:Y84	C:Y85
1	1	4	1	0	2	0	2	0	0	3	0	2

Data for Figure 4B Corneal Scarring

	C:Y86	C:Y87	C:Y88	C:Y89	C:Y90	C:Y91	C:Y92	C:Y93	C:Y94	C:Y95	C:Y96	C:Y97
1	0	0	0	1.5	3	0	0	1.5	3	2.5	0	1.5

Data for Figure 4B Corneal Scarring

	C:Y98	C:Y99	C:Y100	C:Y101	C:Y102	C:Y103	C:Y104	C:Y105	C:Y106	C:Y107	C:Y108	C:Y109
1	4	1	4	0	0	3.5	0	3	0	3	4	0

Data for Figure 4B Corneal Scarring

	C:Y110	C:Y111	C:Y112	C:Y113	C:Y114	C:Y115	C:Y116	C:Y117	C:Y118	C:Y119	C:Y120	C:Y121
1	0	0	0.75	1	4	1	1	0	1	3	1	0

Data for Figure 4B Corneal Scarring

	C:Y122	C:Y123	C:Y124	C:Y125	C:Y126	C:Y127	C:Y128	C:Y129	C:Y130	C:Y131	C:Y132	C:Y133
1	2	1.5	1	4	4	4	1	1.5	4	0.75	4	1.5

Data for Figure 4B Corneal Scarring

	C:Y134	C:Y135	C:Y136	C:Y137	C:Y138	C:Y139	C:Y140	C:Y141	C:Y142	C:Y143
1	1	0.5	2	0.75	2	3	1	2.5		

Data for Figure 4B_Corneal Scarring

Table format: **XY**		X											
		X Title											
		X	A:Y1	A:Y2	A:Y3	A:Y4	A:Y5	A:Y6	A:Y7	A:Y8	A:Y9	A:Y10	A:Y11
1	Title		9	16	6	6	0	0	16	9	16	5	2

Data for Figure 4C_Angiogenesis

	A:Y12	A:Y13	A:Y14	A:Y15	A:Y16	A:Y17	A:Y18	A:Y19	A:Y20	A:Y21	A:Y22	A:Y23
1	2	16	11	4	2	4	4	4	6	0	16	16

Data for Figure 4C_Angiogenesis

	A:Y24	A:Y25	A:Y26	A:Y27	A:Y28	A:Y29	A:Y30	A:Y31	A:Y32	A:Y33	A:Y34	A:Y35
1	2	16	4	6	16	4	2	7	0	9	0	16

Data for Figure 4C_Angiogenesis

	A:Y36	A:Y37	A:Y38	A:Y39	A:Y40	A:Y41	A:Y42	A:Y43	A:Y44	A:Y45	A:Y46	A:Y47
1	4	4	16	6	16	16	2	2	4	16	5	4

Data for Figure 4C_Angiogenesis

	A:Y48	A:Y49	A:Y50	A:Y51	A:Y52	A:Y53	A:Y54	A:Y55	A:Y56	A:Y57	A:Y58	A:Y59
1	16	6	4	0	16	4	2	6	4	4	5	16

Data for Figure 4C_Angiogenesis

	A:Y60	A:Y61	A:Y62	A:Y63	A:Y64	A:Y65	A:Y66	A:Y67	A:Y68	A:Y69	A:Y70	A:Y71
1	4	4	16	6	16	10	16	6	10	16	16	2

Data for Figure 4C_Angiogenesis

	Group A											
	McKrae											
	A:Y72	A:Y73	A:Y74	A:Y75	A:Y76	A:Y77	A:Y78	A:Y79	A:Y80	A:Y81	A:Y82	A:Y83
1	0	0	0	0	0	0	16	0	16	2	1	0

Data for Figure 4C_Angiogenesis

	A:Y84	A:Y85	A:Y86	A:Y87	A:Y88	A:Y89	A:Y90	A:Y91	A:Y92	A:Y93	A:Y94	A:Y95
1	2	0	0	2	0	16	0	0	0	0	0	14

Data for Figure 4C_Angiogenesis

	A:Y96	A:Y97	A:Y98	A:Y99	A:Y100	A:Y101	A:Y102	A:Y103	A:Y104	A:Y105	A:Y106	A:Y107
1	0	2	0	0	0	0	16	16	3	2	0	4

Data for Figure 4C_Angiogenesis

	A:Y108	A:Y109	A:Y110	A:Y111	A:Y112	A:Y113	A:Y114	A:Y115	A:Y116	A:Y117	A:Y118	A:Y119
1	0	3	0	14	6	2	10	16	6	10	0	12

Data for Figure 4C_Angiogenesis

	A:Y120	A:Y121	A:Y122	A:Y123	A:Y124	A:Y125	A:Y126	A:Y127	A:Y128	A:Y129	A:Y130	A:Y131
1	12	0	0	16	6	16	8	6	0			

Data for Figure 4C_Angiogenesis

	A:Y132	A:Y133	A:Y134	A:Y135	A:Y136	A:Y137	A:Y138	A:Y139	A:Y140	A:Y141	A:Y142	A:Y143
1												

Data for Figure 4C_Angiogenesis

	B:Y1	B:Y2	B:Y3	B:Y4	B:Y5	B:Y6	B:Y7	B:Y8	B:Y9	B:Y10	B:Y11	B:Y12
1	2	2	2	4	1	2	2	0	2	2	0	2

Data for Figure 4C_Angiogenesis

	B:Y13	B:Y14	B:Y15	B:Y16	B:Y17	B:Y18	B:Y19	B:Y20	B:Y21	B:Y22	B:Y23	B:Y24
1	2	6	2	2	2	2	2	2	2	6	4	2

Data for Figure 4C_Angiogenesis

	B:Y25	B:Y26	B:Y27	B:Y28	B:Y29	B:Y30	B:Y31	B:Y32	B:Y33	B:Y34	B:Y35	B:Y36
1	16	3	4	0	16	12	7	16	8	16	2	2

Data for Figure 4C_Angiogenesis

	B:Y37	B:Y38	B:Y39	B:Y40	B:Y41	B:Y42	B:Y43	B:Y44	B:Y45	B:Y46	B:Y47	B:Y48
1	0	16	0	16	16	16	16	10	3	16	16	16

Data for Figure 4C_Angiogenesis

	B:Y49	B:Y50	B:Y51	B:Y52	B:Y53	B:Y54	B:Y55	B:Y56	B:Y57	B:Y58	B:Y59	B:Y60
1	16	2	16	16	9	4	16	16	0	2	4	6

Data for Figure 4C_Angiogenesis

	Group B											
	ΔsncRNA1&2											
	B:Y61	B:Y62	B:Y63	B:Y64	B:Y65	B:Y66	B:Y67	B:Y68	B:Y69	B:Y70	B:Y71	B:Y72
1	4	2	3	16	2	2	4	1	6	4	4	16

Data for Figure 4C_Angiogenesis

	B:Y73	B:Y74	B:Y75	B:Y76	B:Y77	B:Y78	B:Y79	B:Y80	B:Y81	B:Y82	B:Y83	B:Y84
1	2	16	2	16	2	0	16	0	0	16	0	0

Data for Figure 4C_Angiogenesis

	B:Y85	B:Y86	B:Y87	B:Y88	B:Y89	B:Y90	B:Y91	B:Y92	B:Y93	B:Y94	B:Y95	B:Y96
1	0	16	0	16	3	2	14	16	0	2	0	0

Data for Figure 4C_Angiogenesis

	B:Y97	B:Y98	B:Y99	B:Y100	B:Y101	B:Y102	B:Y103	B:Y104	B:Y105	B:Y106	B:Y107	B:Y108
1	0	16	16	2	16	16	7	2	0	0	16	16

Data for Figure 4C_Angiogenesis

	B:Y109	B:Y110	B:Y111	B:Y112	B:Y113	B:Y114	B:Y115	B:Y116	B:Y117	B:Y118	B:Y119	B:Y120
1	7	0	0	0	2	4	10	6	14	4	4	4

Data for Figure 4C_Angiogenesis

	B:Y121	B:Y122	B:Y123	B:Y124	B:Y125	B:Y126	B:Y127	B:Y128	B:Y129	B:Y130	B:Y131	B:Y132
1	14	16	0	16	16	6	0	16				

Data for Figure 4C_Angiogenesis

	B:Y133	B:Y134	B:Y135	B:Y136	B:Y137	B:Y138	B:Y139	B:Y140	B:Y141	B:Y142	B:Y143	C:Y1
1												2

Data for Figure 4C_Angiogenesis

	C:Y2	C:Y3	C:Y4	C:Y5	C:Y6	C:Y7	C:Y8	C:Y9	C:Y10	C:Y11	C:Y12	C:Y13
1	2	0	2	2	3	2	16	2	13	2	16	2

Data for Figure 4C_Angiogenesis

	C:Y14	C:Y15	C:Y16	C:Y17	C:Y18	C:Y19	C:Y20	C:Y21	C:Y22	C:Y23	C:Y24	C:Y25
1	2	0	16	16	2	2	16	16	5	16	16	2

Data for Figure 4C_Angiogenesis

	C:Y26	C:Y27	C:Y28	C:Y29	C:Y30	C:Y31	C:Y32	C:Y33	C:Y34	C:Y35	C:Y36	C:Y37
1	16	0	12	2	12	16	16	16	16	10	10	8

Data for Figure 4C_Angiogenesis

	C:Y38	C:Y39	C:Y40	C:Y41	C:Y42	C:Y43	C:Y44	C:Y45	C:Y46	C:Y47	C:Y48	C:Y49
1	8	0	16	16	16	16	16	7	16	0	0	16

Data for Figure 4C_Angiogenesis

	C:Y50	C:Y51	C:Y52	C:Y53	C:Y54	C:Y55	C:Y56	C:Y57	C:Y58	C:Y59	C:Y60	C:Y61
1	16	16	16	2	16	4	4	4	7	4	2	2

Data for Figure 4C_Angiogenesis

	Group C											
	dLAT2903											
	C:Y62	C:Y63	C:Y64	C:Y65	C:Y66	C:Y67	C:Y68	C:Y69	C:Y70	C:Y71	C:Y72	C:Y73
1	4	8	6	4	3	2	2	4	6	4	6	6

Data for Figure 4C_Angiogenesis

	C:Y74	C:Y75	C:Y76	C:Y77	C:Y78	C:Y79	C:Y80	C:Y81	C:Y82	C:Y83	C:Y84	C:Y85
1	8	2	2	6	16	0	0	0	0	5	0	0

Data for Figure 4C_Angiogenesis

	C:Y86	C:Y87	C:Y88	C:Y89	C:Y90	C:Y91	C:Y92	C:Y93	C:Y94	C:Y95	C:Y96	C:Y97
1	16	0	0	0	2	6	16	0	2	0	2	0

Data for Figure 4C_Angiogenesis

	C:Y98	C:Y99	C:Y100	C:Y101	C:Y102	C:Y103	C:Y104	C:Y105	C:Y106	C:Y107	C:Y108	C:Y109
1	5	0	0	5	0	5	0	0	0	0	2	0

Data for Figure 4C_Angiogenesis

	C:Y110	C:Y111	C:Y112	C:Y113	C:Y114	C:Y115	C:Y116	C:Y117	C:Y118	C:Y119	C:Y120	C:Y121
1	0	0	6	5	8	0	0	0	2	2	0	2

Data for Figure 4C_Angiogenesis

	C:Y122	C:Y123	C:Y124	C:Y125	C:Y126	C:Y127	C:Y128	C:Y129	C:Y130	C:Y131	C:Y132	C:Y133
1	5	6	3	16	16	16	4	5	16	2	16	3

Data for Figure 4C_Angiogenesis

	C:Y134	C:Y135	C:Y136	C:Y137	C:Y138	C:Y139	C:Y140	C:Y141	C:Y142	C:Y143
1	4	2	6	3	6	8	4	7		

Data for Figure 4C_Angiogenesis

Table format: **Column**		Group A	Group B	Group C	Group D	Group E
		McKrae	Data Set-B	ΔsncRNA1&2	Title	dLAT2903
1	Title	4		4		6
2	Title	4		4		6
3	Title	4		4		9
4	Title	4		4		6
5	Title	4		4		9
6	Title	4		4		9
7	Title	4		4		9
8	Title	4		5		6
9	Title	4		5		9
10	Title	4		5		8
11	Title	5				9
12	Title	5				5
13	Title	6		4		9
14	Title			4		8
15	Title	4		4		8
16	Title	4		4		5
17	Title	5		4		8
18	Title	5		4		5
19	Title	5		4		8
20	Title	5		4		8
21	Title	5		4		
22	Title	6		4		
23	Title	6		4		
24	Title	6		4		
25	Title			5		
26	Title			5		
27	Title			5		
28	Title			5		
29	Title			5		
30	Title	3		3		4
31	Title	3		3		4

Data for Figure 5A_Reactivation

Data for Figure 5A_Reactivation

Table format: **Column**		Group A	Group B	Group C	Group D	Group E
		McKrae	Data Set-B	ΔsncRNA1&2	Title	dLAT2903
32	Title	4		3		4
33	Title	4		3		4
34	Title	4		3		4
35	Title	4		4		4
36	Title	4		4		4
37	Title	4		4		4
38	Title	5		4		5
39	Title	6		4		5
40	Title	7		4		6
41	Title			4		

Table format: **Grouped**												
		A:1	A:2	A:3	A:4	A:5	A:6	A:7	A:8	A:9	A:10	A:11
1	Title	1374848.578	728096.043	1657335.831	1119750.250	1002506.246	3031264.799	274323.157	1521597.604	1984769.190	1287722.144	1035197.414

Data for Figure 5B_gB DNA copy#

	Group A											
	McKrae											
	A:12	A:13	A:14	A:15	A:16	A:17	A:18	A:19	A:20	A:21	A:22	A:23
1	1862648.634	356945.819	969541.378	2403598.000	4741895.000	1153131.000	1284796.000	1581992.000	348439.000	1145655.000	949073.900	1687186.000

Data for Figure 5B gB DNA copy#

	A:24	A:25	A:26	A:27	A:28	A:29	A:30	A:31	A:32	A:33	A:34	A:35
1	2653161.000	1113900.000	2858148.000	2634926.000	798589.900	2622754.000	2404432.000	1070780.000	1142209.000			

Data for Figure 5B gB DNA copy#

	A:36	A:37	A:38	A:39	A:40	B:1	B:2	B:3	B:4	B:5	B:6
1						653003.7899	600313.9602	876883.0328	877553.9412	1344091.6840	402417.5701

Data for Figure 5B gB DNA copy#

	B:7	B:8	B:9	B:10	B:11	B:12	B:13	B:14	B:15	B:16	B:17
1	1010511.5250	1769900.6680	788366.2755	1459923.6070	1369638.3680	940775.6270	1385707.0000	162152.4000	1065372.0000	1220572.0000	467872.2000

Data for Figure 5B gB DNA copy#

	Group B										
	ΔsncRNA1&2										
	B:18	B:19	B:20	B:21	B:22	B:23	B:24	B:25	B:26	B:27	B:28
1	1613087.0000	2972839.0000	1010930.0000	1679710.0000	470787.3000	2445026.0000	0.0000	4870.8260	276102.7000	2680539.0000	375832.1000

Data for Figure 5B gB DNA copy#

	B:29	B:30	B:31	B:32	B:33	B:34	B:35	B:36	B:37	B:38	B:39
1	1712640.0000	1587316.0000									

Data for Figure 5B gB DNA copy#

	B:40	C:1	C:2	C:3	C:4	C:5	C:6	C:7	C:8	C:9	C:10
1		127782.6288	530491.7274	156249.6420	1187495.0600	317824.4000	1146534.8340	288462.6183	1613701.7180	1302325.8110	752764.5332

Data for Figure 5B gB DNA copy#

	Group C										
	dLAT2903										
	C:11	C:12	C:13	C:14	C:15	C:16	C:17	C:18	C:19	C:20	C:21
1	1243069.8820	966844.2961	2257604.0020	29301.1769	34163.2900	1303142.0000	81333.1200	41979.0900	2803185.0000	2720103.0000	314142.4000

Data for Figure 5B_gB DNA copy#

	C:22	C:23	C:24	C:25	C:26	C:27	C:28	C:29	C:30	C:31	C:32
1	69056.5400	83273.7400	102174.3000	2285612.0000	610182.9000	2227944.0000	85812.0800				

Data for Figure 5B gB DNA copy#

	C:33	C:34	C:35	C:36	C:37	C:38	C:39	C:40
1								

Data for Figure 5B_gB DNA copy#

Table format: **Grouped**									
		A:1	A:2	A:3	A:4	A:5	A:6	A:7	A:8
1	Title	272476.92	448766.16	3314760.30	2007424.70	1510329.60	315458.30	1740326.10	27037.93

Data for Figure 6A LAT copy# in TG

	A:9	A:10	A:11	A:12	A:13	A:14	A:15	A:16	A:17
1	993527.87	1123639.70	49651.43	201283.48	1057127.00	836111.57	710162.00	44739.08	1490542.57

Data for Figure 6A LAT copy# in TG

	A:18	A:19	A:20	A:21	A:22	A:23	A:24	A:25	A:26
1	871165.74	218955.29	851278.11	6.55e+008	2.29e+008	1.87e+009	1.59e+009	2.14e+008	1.79e+009

Data for Figure 6A LAT copy# in TG

	Group A								
	McKrae								
	A:27	A:28	A:29	A:30	A:31	A:32	A:33	A:34	A:35
1	1.56e+009	6.47e+009	2.15e+008	5.39e+009	1.88e+009	3.86e+008	2.00e+009	7.94e+009	9.09e+009

Data for Figure 6A LAT copy# in TG

	A:36	A:37	A:38	A:39	A:40	A:41	A:42	A:43	A:44
1	4.01e+009	6.68e+009	4.06e+009	1.05e+010	5.62e+009	1.38e+010	4.90e+009	5.98e+009	7.64e+009

Data for Figure 6A LAT copy# in TG

	A:45	A:46	A:47	A:48	A:49	A:50	A:51	A:52	A:53
1	3.04e+009	5.96e+008	6.10e+009	2.15e+010	1.63e+010	2.52e+010			

Data for Figure 6A LAT copy# in TG

	A:54	A:55	B:1	B:2	B:3	B:4	B:5	B:6	B:7
1			1294395	55382	4572475	3537222	1160750	50001	1979691

Data for Figure 6A LAT copy# in TG

	B:8	B:9	B:10	B:11	B:12	B:13	B:14	B:15	B:16
1	2588634	1215539	1668266	317435	846112	1962605	363647	2672069	1374385

Data for Figure 6A LAT copy# in TG

	B:17	B:18	B:19	B:20	B:21	B:22	B:23	B:24	B:25
1	1040589	118854	324904	1010757	1428948	605446	134728	3e+009	7e+009

Data for Figure 6A LAT copy# in TG

	Group B ΔsncRNA1&2								
	B:26	B:27	B:28	B:29	B:30	B:31	B:32	B:33	B:34
1	2e+010	1e+010	9e+009	6e+009	6e+009	9e+009	8e+009	1e+010	2e+010

Data for Figure 6A LAT copy# in TG

	B:35	B:36	B:37	B:38	B:39	B:40	B:41	B:42	B:43
1	2e+010	3e+010	2e+010	2e+010	1e+010	2e+010	2e+010	3e+010	5e+009

Data for Figure 6A LAT copy# in TG

	B:44	B:45	B:46	B:47	B:48	B:49	B:50	B:51	B:52
1	4e+010	2e+010	9e+009	3e+010	2e+010	3e+010			

Data for Figure 6A_LAT copy# in TG

	B:53	B:54	B:55
1			

Data for Figure 6A_LAT copy# in TG

Table format: **Grouped**		Group A McKrae			Group B ΔsncRNA1&2		
		A:1	A:2	A:3	B:1	B:2	B:3
1	12	783100.1900	1381342.300	1335890.590	7070122.950	8384506.760	7699317.750
2	24	7.398810063	1.081404326	1.958002965	4.491973615	3.293251759	3.079439043
3	48	4.330859586	3.609599898	5.025007011	6.847714102	1.190241803	7.004140384

Data for Figure 6B_ LAT copy# in Neuro 2A

Table format: **Grouped**		Group A Mock						Group B McKrae				
		A:1	A:2	A:3	A:4	A:5	A:6	B:1	B:2	B:3	B:4	B:5
1	Title	7.12	2.68	5.00	2.96	2.76	2.53	44	47	47	23	19

Data for Figure 7B_cleaved Caspase 3

		Group C						Group D				
		sncRNA1&2						dLAT2903				
	B:6	C:1	C:2	C:3	C:4	C:5	C:6	D:1	D:2	D:3	D:4	D:5
1	11	55.4	63.1	59.3	57.1	59.3	59.1	58.6	62.6	60.5	58.5	47.0

Data for Figure 7B_cleaved Caspase 3

	D:6
1	58.7

Data for Figure 7B_cleaved Caspase 3
